# Haplotype-based Noninvasive Prenatal Diagnosis of Hyperphenylalaninemia through Targeted Sequencing of Maternal Plasma

**DOI:** 10.1038/s41598-017-18358-y

**Published:** 2018-01-09

**Authors:** Jun Ye, Chao Chen, Yuan Yuan, Lianshu Han, Yaoshen Wang, Wenjuan Qiu, Huiwen Zhang, Asan, Xuefan Gu

**Affiliations:** 1 Department of Pediatric Endocrinology and Genetic Metabolism, Shanghai Institute for Pediatric Research, Xinhua Hospital, Shanghai Jiao Tong University School of Medicine, Shanghai, 200092 China; 2 Tianjin Translational Genomics Center, BGI-Tianjin, BGI-Shenzhen, Tianjin, 300308 China; 3 Binhai Genomics Institute, BGI-Tianjin, BGI-Shenzhen, Tianjin, 300308 China; 4 BGI-Shenzhen, Shenzhen, China

## Abstract

Here we developed a haplotype-based noninvasive prenatal diagnosis method for hyperphenylalaninemia (HPA) and demonstrated its accuracy and feasibility during early pregnancy. Capture sequencing was performed on genomic DNA from parents and probands using customized hybridization probes targeting highly heterozygous single-nucleotide polymorphisms located within the 1 M region flanking phenylalanine hydroxylase (*PAH*) and 6-pyruvoyltetrahydropterin (*PTS*) and its coding region to determine the parental haplotypes and linkage to pathogenic mutations. Maternal plasma DNA obtained at 12–20 weeks of gestation was also subjected to targeted sequencing to deduce the fetal haplotypes based on the parental haplotypes. The fetal genotypes were further validated by invasive prenatal diagnosis. Haplotype-based noninvasive prenatal testing was successfully performed in 13 families. Five fetuses were identified to harbor bi-allelic pathogenic variants of PAH, four fetuses were carriers of one heterozygous PAH variant, three fetuses were normal, and the fetus of the 6-pyruvoyl tetrahydrobiopterin synthase family was identified as normal. The fetal genotypes at two gestational weeks from the same PAH family were identical. All results were consistent with the prenatal diagnosis based on amniotic fluid. Haplotype-based noninvasive prenatal testing for HPA through targeted sequencing is accurate and feasible during early gestation.

## Introduction

Hyperphenylalaninemia (HPA) is one of the most common autosomal recessive amino acid metabolic diseases with a prevalence of 1/10,397 in China according to 35 million newborn screening data^1–4^. HPA is mainly caused by phenylalanine hydroxylase (PAH) deficiency resulting primarily from pathogenic variants in *PAH (NM_000277.1)* or PAH cofactor tetrahydrobiopterin deficiency^5^, which accounts for approximately 12.9% of individuals with HPA in China^6^. Tetrahydrobiopterin deficiency mainly occurs because of 6-pyruvoyl tetrahydrobiopterin synthase (PTPS) deficiency caused by mutations of 6-pyruvoyltetrahydropterin synthase (*PTS, NM_000317.2*) (accounting for 96% in China)^7^, which can be distinguished from PAH deficiency by urinary pterin analysis. HPA is a treatable metabolic disorder; early diagnosis and early treatment by newborn screening can prevent neurological damage and permanent intellectual disability. But some patients had poor disease control because of expensive treatment costs and lack of professional diet guidance. Prenatal diagnosis as early as possible is very helpful for high-risk families considering that potential medical management is available. However, invasive procedures have a small miscarriage risk and strict time-control problem^8,9^. The discovery of cell-free fetal DNA (cff-DNA) in the maternal plasma generated new expectations for a noninvasive prenatal test (NIPT). Polymerase chain reaction (PCR)-based noninvasive prenatal test cannot be applied to detect recessive inherited diseases, as it can only detect pathogenic alleles discriminated from the maternal allele^10,11^.

Technological developments in massively parallel sequencing have accelerated the development of NIPT technology. In addition to its wide application in detecting fetal aneuploidy^12,13^, NIPT of recessive inherited single-gene diseases has also been achieved using maternal plasma sequencing and haplotype analysis^14,15^. However, no studies have examined NIPT of HPA. In this study, we performed haplotype-assisted NIPT of HPA for 13 singleton pregnant women with a reproductive history of an HPA-affected child (12 PAH deficiency and 1 PTS deficiency) using targeted sequencing data of maternal plasma and samples of the trio (parents and the proband). The purpose of this study was to assess the accuracy and feasibility of haplotype-assisted NIPT of HPA and explore its potential utilization in the first trimester.

## Results

### Sequencing data and measurements of cff-DNA fraction

A mean depth of 213.27X (77.27X–356.07X) was obtained in the genomic DNA samples and 358.48X (202.40X–590.63X) in the plasma DNA samples. Approximately 99.69% (97.74–99.96%) of the targeted region was covered by more than 20 reads. The mean capture specificity was 50.33% (32.51–71.51%) in all samples. The mean duplicate rate was 14.50% (1.44–36.23%) in genomic DNA, while the mean sequencing duplicate rate of plasma samples was 51.01% (20.58–75.24%) (Supplemental Table S1). The higher duplicate read rates were caused by additional PCR cycles during library preparation and target enrichment because of the low amount of plasma DNA. The mean sequencing error rate was 0.0953% (0.0404–0.2696%) (Table 1). These data showed high experimental quality for subsequent analysis.

**Table 1:** Statistics of Fetal DNA Fractions and Error Rate.

Pedigree	Type 1 SNP^a^	Type 2 SNP^b^	Error Rate	Fetal DNA fraction
F01	14	186	0.0912%	8.13%
F02	9	178	0.0428%	6.70%
F03	8	38	0.0416%	10.65%
F04	17	124	0.0419%	6.60%
F06	12	104	0.1034%	4.91%
F07	4	113	0.0494%	20.07%
F08-12GW	18	126	0.1626%	7.29%
F08-18GW	14	125	0.1493%	9.00%
F09	11	80	0.0538%	6.38%
F10	6	130	0.0425%	18.99%
F11-12GW	21	119	0.1125%	7.37%
F11-17GW	16	140	0.0641%	7.73%
F12	27	94	0.0971%	7.83%
F13-12GW	31	173	0.0685%	5.79%
F13-16GW	25	179	0.1809%	7.34%
F05	16	40	0.2696%	6.94%

SNP^a^ that were homozygous with the same genotype in both parental genomes but had different bases in the plasma and were used to calculate the sequencing error rate of plasma; SNP^b^ that were homozygous in both parents but had different types and were used to calculate cff-DNA fractions.

The cff-DNA fraction of 13 HPA families varied from 4.52% to 20.43% (Table 1), showing significant differences between individuals. The cff-DNA fractions at 12 weeks of gestation in F08, F11, and F13 family were slightly lower than those at 16, 17, and 16 weeks of gestation respectively, while there were no significant differences between two time points.

### Noninvasive prenatal testing of HPA

**Figure 1:**
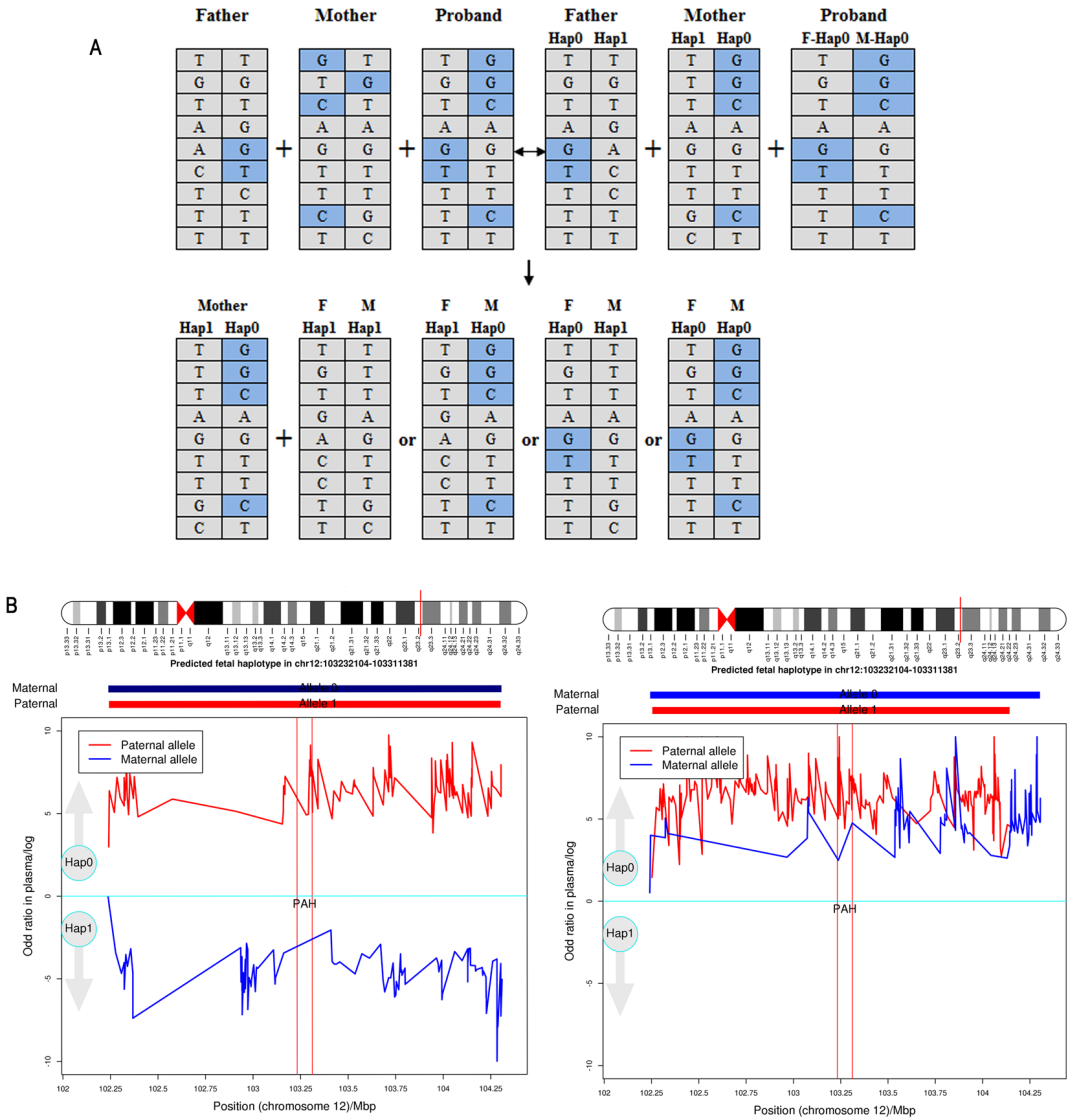
Fetal Haplotype Prediction. (**A**) Schematic diagram of trio phasing and haplotype recovery. (**B**) Probability of fetal inherited haplotype. X-axis represents the locus on chromosome 12; Y-axis represents the logarithm of the ratios of different fetal haplotype combinations. Red lines represent the fetus-inherited paternal haplotype; blue represents line fetus inherited from the maternal haplotypes. The lines above zero (cyan lines) indicate that the fetus inherited the pathogenic allele (Hap0), and the lines below zero indicate that the fetus inherited the benign allele (Hap1). Left chart and right chart represent the NIPT results of families F01 and F02, respectively.

The number of SNPs identified in the target region for NIPT ranged from 746 to 1614 for each sample. Parental haplotypes were successfully constructed using the SNPs of the proband and parents in the *PAH* or *PTS* target region. The average number of informative SNPs were used to predict the fetus-inherited maternal haplotype and paternal haplotype of 139 (range: 72–322) and 158 (range: 32–418), respectively. HMM-based prediction of the fetal haplotype is shown in Fig. 1A. Using the F02 family as an example, parental haplotypes across the *PAH* coding and flanking 1 M region were constructed. The results showed that the number of SNPs supporting that the fetus inherited the pathogenic allele from the father was 207 and none supported the normal haplotype, while the number of SNPs supporting that the fetus inherited the pathogenic allele from the mother was 88 and none supported the normal haplotype. Thus, the fetus in the F02 family was affected by HPA because of the F0 + M0 haplotype combination (Table 2 and Fig. 1B). Based on this strategy, the fetuses in F02, F08, F09, F12, and F13 were affected by HPA, fetuses in F01, F04, F07, and F10 were diagnosed as carriers, and fetuses in F03, F05, F06, and F11 were normal (Table 2 and Supplemental Fig. S1). These data are consistent with the results of invasive procedures (Table 2).

**Table 2:** Haplotype-based NIPT Results of Studied Families.

Family	Gene	SNPs For F0^a^	SNPs For F1^a^	SNPs For M0^a^	SNPs For M1^a^	Fetal Haplotype	Fetal Genotype	NIPT Results	Invasive Test Results	Consistency^b^
F01	PAH	117	0	0	98	F0 + M1	c.728 G > A/N	Carrier	c.728 G > A/N	Y^c^
F02	PAH	207	0	88	0	F0 + M0	c.764 T > C/c.1197 A > T	affected	c.764 T > C/c.1197 A > T	Y
F03	PAH	18	87	0	84	F1 + M1	N	Normal	N	Y
F04	PAH	125	0	0	152	F0 + M1	c.770 G > T/N	Carrier	c.770 G > T/N	Y
F06	PAH	0	79	0	132	F1 + M1	N	Normal	N	Y
F07	PAH	0	184	91	0	F1 + M0	c.977 G > A/N	Carrier	c.977 G > A/N	Y
F08-12GW	PAH	105	0	119	0	F0 + M0	c.838 G > A/c.728 G > A	affected	c.838 G > A/c.728 G > A	Y
F08-18GW	PAH	60	0	72	0	F0 + M0	c.838 G > A/c.728 G > A	affected	c.838 G > A/c.728 G > A	Y
F09	PAH	181	0	189	0	F0 + M0	c.208_210delTCT/c.473 G > A	affected	c.208_210delTCT/c.473 G > A	Y
F10	PAH	0	169	99	0	F1 + M0	c.1045 T > G/N	Carrier	c.1045 T > G/N	Y
F11-12GW	PAH	10	50	0	125	F1 + M1	N	Normal	N	Y
F11-17GW	PAH	21	115	0	143	F1 + M1	N	Normal	N	Y
F12	PAH	139	0	142	36	F0 + M0	c.764 T > C/c.611 A > G	affected	c.764 T > C/c.611 A > G	Y
F13-12GW	PAH	418	0	322	0	F0 + M0	c.721 C > T/c.728 G > A	affected	c.721 C > T/c.728 G > A	Y
F13-16GW	PAH	418	0	322	0	F0 + M0	c.721 C > T/c.728 G > A	affected	c.721 C > T/c.728 G > A	Y
F05	PTS	0	32	0	94	F1 + M1	N	Normal	N	Y

^a^SNPs for F/M represents the SNP number that supported fetal inherited from paternal haplotypes. F0/M0, fetal-inherited parental mutant haplotype; F1/M1, fetal-inherited paternal wild-type haplotype; ^b^Consistency represents comparison the NIPT results with invasive testing results; ^c^Y, yes; N, normal.

The fetal haplotypes predicted at 12 weeks of gestation in F08, F11, and F13 were accordant with those predicted at 16–17 weeks of gestation (Table 2 and see Supplemental Fig. S1).

### Accuracy of haplotype-based NIPT for HPA

The fetal genotype in *PAH and PTS* deduced by NIPT were consistent with that obtained by the invasive procedure. The performance of NIPT was further evaluated using SNPs in the target region of DNA from amniotic fluid. SNPs deduced by predicted parental haplotype inheritance based on a trio strategy were 100% consistent with the fetal DNA sequencing data in 13 families (Supplemental Table S2).

### Assessment of feasibility of NIPT in first-trimester

First, we evaluated the relationship between the sequencing depth and the accuracy of the inferred fetal haplotype, meanwhile the variable factor fetal fraction was assigned 10%. (Supplemental Table S3 and Supplemental Fig. S2). If 20 SNPs was used to inferred fetal inherited maternal haplotype to reach 99% detection accuracy, the sequence depth of plasma must achieve 100X. If the sequence depth of plasma achieved 200X, We only need about 10 SNPs to predict the fetal inherited maternal haplotype with 99% detection accuracy.

Second, we evaluated relationship between fetal fraction/informative SNPs and the accuracy of the inferred maternal fetal SNPs. The simulated data showed that when the fetal fraction is 8%, 9% and 10%, the corresponding SNPs required for fetal haplotype constructing are ~20, ~12, and ~10 to reach 99% detection accuracy of maternal alleles of the fetus. (Supplemental Table S4 and Supplemental Fig. S3).

## Discussion

Noninvasive prenatal diagnosis of genetic disorders has become a new trend in clinical practice since the discovery of cff-DNA in the maternal plasma. Prenatal diagnosis as early as possible is very helpful for high-risk families considering that potential medical management is available. However, the gestational week in most reported research of NIPD are in the second trimester or in early pregnancy but lack system evaluation. In this study, we demonstrated the feasibility and evaluated the accuracy of NIPD of HPA before 12 gestational week. Based on the relationship between the sequencing depth and the accuracy of the inferred fetal haplotype using computer simulation, the cutoff value of the depth of plasma was determined to be 200X due to only about 10 SNPs (Supplemental Table S3 and Fig. S2) to predict the fetal inherited maternal haplotype with 99% accuracy. In addition to the depth simulation, we evaluated relationship between fetal fraction/informative SNPs and the accuracy of the inferred maternal fetal SNPs. The accuracy was affected by the fetal fraction lower than 5%, when the fetal fraction is between 5% and 10%, the mean number of SNP is about 20 to reach 99% detection accuracy. The minimum number of informative SNP for fetal maternal haplotype constructing in F08-18GW family is 72. The number of informative SNPs of three families at early gestation in this study for fetal maternal haplotype constructing are all above 100. The fetal DNA fraction at 10th gestational week was about 9% and the total increment of median fetal fraction from 5th to 12th gestational week was about just 1%^16,17^. When the plasma sequence depth is 200X, the accuracy of fetal inherited maternal haplotype is all above 99% and these data could prove our method to be feasibility in the first trimester. To validate the conclusion of simulated data, we collected plasma both at 12 weeks and 16–17 weeks of gestation for F08, F11, and F13. The fetal haplotypes predicted at two gestational ages were completely consistent. The concentration of fetal DNA slightly increased at later gestational age, but showed no significant difference between two times. The fetal fractions were 7.29%, 7.37%, and 5.79% at 12 weeks of gestation in F08, F11, and F13, respectively; the fetal fractions were 9.08% and 7.34% at 16 weeks in F08 and F13; and the fetal fraction was 7.73% at 17 weeks in F11. In F06, the fetal DNA fraction at 20 weeks of gestation was only 4.91% lower than the cutoff value 5%, however, there was enough 132 SNPs supported to predict the fetal inherited maternal haplotype with 100% accuracy. Our study laid the foundation for further prospective studies.

The target region previously reported is relatively large and the sequencing cost is exorbitant. In order to optimize the cost of NIPT of monogenic disease, we could narrows the capture region. In this study, 516.27 kb customized hybridization probes including the coding region and highly heterozygous SNPs located within the 1 M region flanking *PAH* or *PTS*. Another path was to find the balance point between maximization of sample on the same probe capture reaction and data utilization rate. In our study, five families’ samples including the genomic DNA (gDNA) of father, mother and proband and maternal cff-DNA are the best experimental scheme. Fifteen gDNA libraries and five plasma DNA libraries are pooled into one library, respectively, and then followed by the same custom-designed NimbleGen SeqCap EZ probes capture procedure. According to this design, the NIPT cost of one family sample is about 4700 RMB. For an urgent family sample, the time of response should take precedence over the cost, the cost is about 6000 RMB due to doing experiments alone.The current NIPT procedure was completed in 7 days including DNA extraction (0.5 days) library construction (one day), library pooling and capture (2.5 days), sequencing (2 days) and data analysis (one day). New technology can shorten this time. A one-tube method for library preparation and customized hybridization probes for capturing would shorten the time from 4 to 2 days before sequencing. The whole test procedure can be further reduced to 4 days or less with advances in sequencing technology.

The haplotype-assisted NIPT of monogenic diseases have been reported, this method has not been applied to HPA. In this study, the fetal genotype in 13 families was successfully constructed using haplotype-assisted NIPT. These results are consistent with invasive method showing an accuracy of 100% except F12. Two SNPs were predicted incorrectly because of a recombination event in the maternal haplotype determination process. The results of NIPT in F12 were credible because the recombination point is far from the pathogenic allele. The proband and the new fetus both have recombination possibility and the approach couldn’t differentiate whether the recombination event occur in the proband or in the new fetus. In a recent study, Yoo *et al*. performed a haplotype-based approach for NIPT of DMD after examining and correcting for the recombination event^18^, the inherited fetal genotype could be also correctly predicted. The probability transfer matrix was constructed through introducing SNP recombination when building HMM model. Thus the interference of the occurrence of recombinant to HMM model prediction is very small, in addition to the detection of recombinant site located at the edge, other recombinant site was validated by the amniotic fluid.

Our group recently developed a haplotype-assisted method for NIPT of recessive monogenic diseases^19^. The fetal genotype was predicted based on whether the haplotype linked to the mutant allele or wild-type allele was inherited. The parental haplotypes were obtained using a trio strategy. An HMM model was built based on the SNP status from the plasma DNA sequencing data and paternal haplotype information to decode the most likely transmitted haplotype using all SNPs in the analysis region. Because errors caused by single-allele analysis can be decreased dramatically, the problem of the maternal background signal was reduced and accurate determination of both paternal and maternal inheritance was obtained. We demonstrated the feasibility of the method utilized in GJB2^14^ and DMD^18^, but this method has not been applied for HPA.

In this study, haplotype-based NIPT was first applied in HPA prenatal testing, which expanded the scope of NIPT application in monogenic disorders and will have profound implications on future prenatal diagnostics. This method can reduce unnecessary invasive procedures as a first-tier screening test. With the development of sequencing technologies, the cost will continue to decrease. NIPT makes genetic testing of the fetus at an early gestational age possible and shows great potential for the diagnostics of monogenic disorders. Additional studies should be conducted to evaluate the usefulness of this technology in clinical practice.

## Materials and Methods

### Patient recruitment and sample collection

Twelve PAH-deficient families and one PTPS-deficient family were recruited at Xin Hua Hospital affiliated with Shanghai Jiao Tong University School of Medicine. Twelve PAH-deficient probands and a PTPS-deficient proband were diagnosed by newborn screening for HPA or based on clinical symptoms such as mental retardation or convulsion and fair hair. Differential diagnosis of etiology was performed by determining urinary pterin and dihydropteridine reductase activity in these probands. Routine Sanger sequencing of *PAH* and *PTS* was performed to identify causative variants. Detailed information of the variants and gestational age are shown in Table 3.

**Table 3:** Molecular Diagnosis Results in 13 Families. Abbreviations: N, normal; GW, gestational weeks.

Family	Gene	Mutation Type			GW
		Father	Mother	Proband	
F01	PAH	c.728 G > A/N	c.728 G > A/N	c.728 G > A/c.728 G > A	17
F02	PAH	c.764 T > C/N	c.1197 A > T/N	c.764 T > C/c.1197 A > T	18
F03	PAH	c.728 G > A/N	c.1045 T > G/N	c.728 G > A/c.1045 T > G	18
F04	PAH	c.770 G > T/N	c.992 T > C/N	c.770 G > T/c.992 T > C	17
F06	PAH	c.611 A > G/N	c.728 G > A/N	c.611 A > G/c.728 G > A	20
F07	PAH	c.1238 G > C/N	c.977 G > A/N	c.1238 G > C/c.977 G > A	17
F08	PAH	c.838 G > A/N	c.728 G > A/N	c.838 G > A/c.728 G > A	12,18
F09	PAH	c.208_210delTCT/N	c.473 G > A/N	c.208_210delTCT/c.473 G > A	18
F10	PAH	c.1197 A > T/N	c.1045 T > G/N	c.1197 A > T/c.1045 T > G	17
F11	PAH	c.727 C > T/N	c.1223 G > A/N	c.727 C > T/c.1223 G > A	12,17
F12	PAH	c.764 T > C/N	c.611 A > G/N	c.764 T > C/c.611 A > G	18
F13	PAH	c.721 C > T/N	c.728 G > A/N	c.721 C > T/c.728 G > A	12,16
F05	PTS	c.116_119delTGTT/N TGTT/N	IVS1-129A > G/N	c.116_119delTGTT/IVS1-129A > G	18

**Figure 2:**
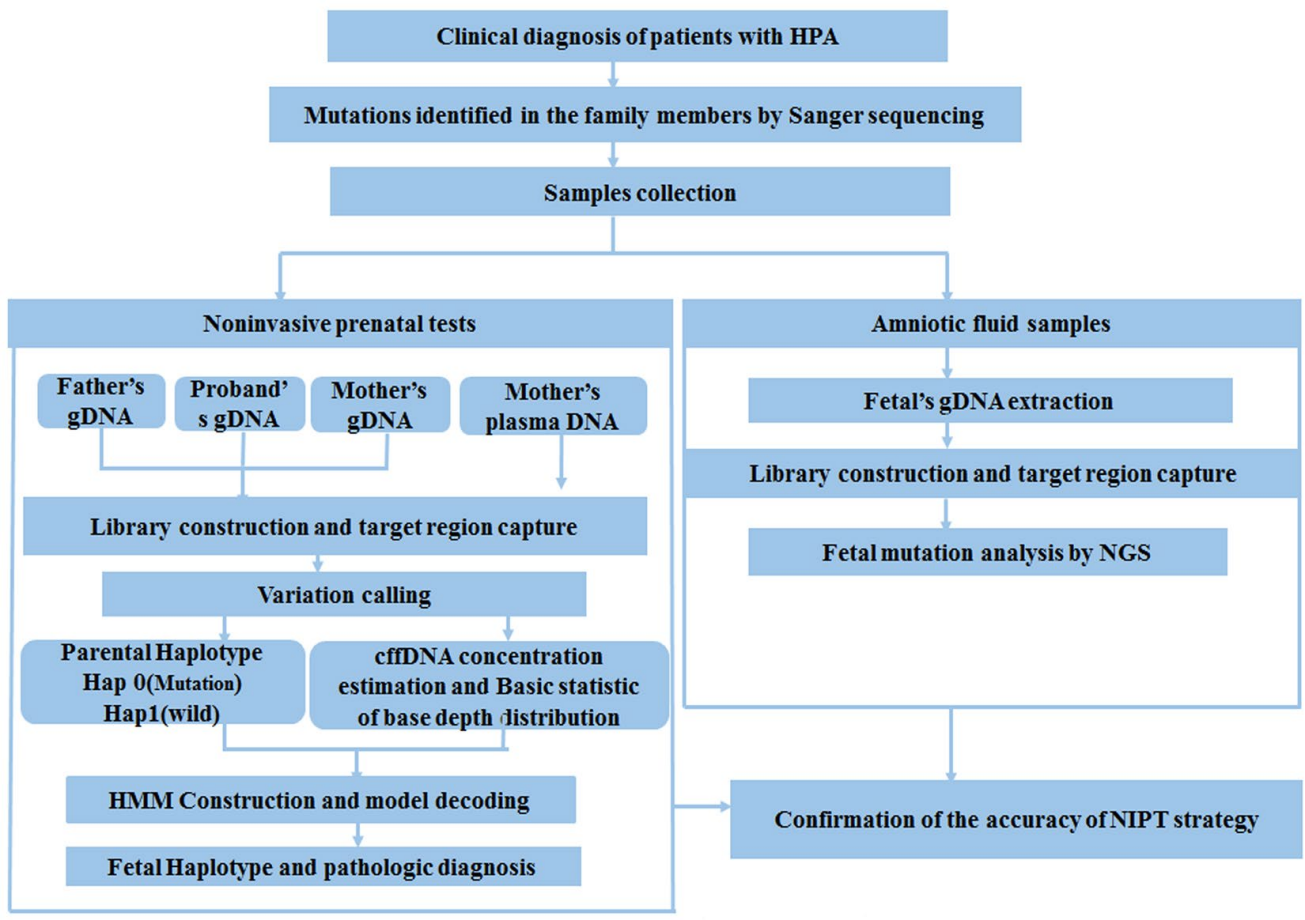
Work Flow of Noninvasive Prenatal Diagnosis. The upper panel is the section of core sample collection whose genotype were identified. The lower panels contains two parallel blind test. The left section is NIPT and the right section is invasive procedure. Finally, the accuracy of this haplotype-based NIPT of HPA was evaluated by amniocentesis prenatal diagnosis.

Blood samples were collected from each pregnant woman, the husband, and their first child and placed into EDTA-containing tubes. Maternal plasma at 16–20 weeks gestation was separated by a two-step centrifugation method within 8 h of collection^20^. Three maternal plasma samples were collected at 12 weeks of gestation to test the feasibility of NIPT in the first trimester. Routine prenatal diagnosis was also performed, amniotic fluid (AF) samples were obtained at 16–18 weeks of gestation, and Sanger sequencing was used to identify mutations as previously described. In plasma DNA analysis, *PAH* and *PTS* mutations in fetuses were blinded to data analysts. The study design is illustrated in Fig. 2.

Informed consent in accordance with the Declaration of Helsinki was obtained from all participants. The study was approved by the ethics committee of Shanghai Xinhua Hospital and BGI (ethics approval number: XHEC-C2014-057 and BGI-IRB 14055-1) and all experimental procedures were performed in accordance with relevant guidelines and regulations.

### Targeted sequencing

Genomic DNA was extracted from 300 μL blood and centrifuged from 10 mL amniotic fluid using the QIAamp DNA Blood Mini Kit (Qiagen, Hilden, Germany), and then 1 μg gDNA was sheared into a target peak size of 200–250 base pairs (bp) using a Covaris S2 (Life Technologies, Carlsbad, CA, USA). After end-repairing and A-tailing processes, adapters were ligated to each end of the DNA fragments, and then the library was enriched and labeled with barcode primers by 4–6 cycles of PCR. Cell-free DNA (cf-DNA) was isolated from 1.8 mL maternal plasma using the QIAamp Circulating Nucleic Acid Kit (Qiagen) and quantified using a Qubit 2.0 fluorometer (Invitrogen, Carlsbad, CA, USA). Next, 10–20 ng cell-free DNA was directly used for library preparation using the KAPA library preparation kit (Kapa Biosystems, Wilmington, MA, USA) because of its highly fragmented nature (approximately 170 bp). After end-repair and “A”-overhanging, adapters were ligated to the DNA fragments, and the library was enriched and labeled with index primers in 8 cycles of PCR.

Twenty gDNA libraries and five cf-DNA libraries were equally pooled to obtain 1 µg of sample. The pooled libraries were captured using the same custom-designed NimbleGen SeqCap EZ probes (Roche NimbleGen, Basel, Switzerland) within the 516.36 kb region, including the *PAH* and *PTS* coding region and 4847 surrounding highly heterozygous single-nucleotide polymorphisms (SNPs) distributed within 1 Mb of chromosome 11 and 12 according to the manufacturer’s instructions and as previously described. The size distribution of the post-capture libraries was analyzed with a Bio-Analyzer 2100 (Agilent Technologies, Santa Clara, CA, USA), quantified by quantitative-PCR (Life Technologies), and subjected to paired-end 101-bp sequencing on an Illumina Hiseq 2500 platform (San Diego, CA, USA).

### Alignment and SNP calling

Paired-end reads were mapped against the human reference genome (Hg19, GRCh37) using BWA software (0.7.12). Picard Tools were used to remove PCR-duplicated and multiple aligned reads. After filtering out low-quality reads with mapping quality < 13 and base quality < 13, SNP calling was performed using GATK software. Variants meeting the following three criteria were used for further analysis: (i) depth greater than 50X, (ii) quality value greater than 30, and (iii) allele frequency larger than 1% by plasma DNA SNP calling.

### Fetal DNA concentration and haplotype-based NIPT of HPA

The fetal DNA fraction was estimated to confirm the existence of fetal cell-free DNA in the plasma sample and assist in the construction of a hidden Markov model (HMM) for NIPT. The fetal DNA fraction in maternal plasma was calculated using SNPs that were homozygous in both parents with different genotype according to the formula: ε = 2d_F_/d_F_ + d_M_, where ε represents the fetal DNA fraction in the plasma and d_F_ and d_M_ represent the depth of the special base of the father and mother, respectively. SNPs that were homozygous in both parents with the same genotype (a) but had different genotypes (b) in the plasma were used to calculate the sequencing error rate (c) using the following equation: c = b/a.

Parental haplotypes and linked pathogenic mutations were constructed using a trio strategy imposed by Mendel’s laws^21^ using SNP information within the 1 M flanking and coding region of *PTS* or *PAH* from the parents and probands. We defined Hap 0 as the haplotype linked to the pathogenic mutation and Hap1 as the haplotype linked to the wild-type allele. Paternal inheritance was determined using SNPs heterozygous in the father but homozygous in the mother. Maternal inheritance was determined using SNPs that were heterozygous in the mother but homozygous in the father. We defined haplotype-informative SNPs as those that were uniquely identified in one haplotype, but not in the other three haplotypes. Informative SNPs linked to the inherited haplotype were over-represented in the plasma. We constructed an HMM to determine the inheritance of parental haplotypes from the plasma data.

Loci that can be used to infer the fetal haplotype in a certain block was denoted as N, while the total number of loci was denoted as n. For each locus, we denoted the observed state as S = {Ni}, where Ni = {Pos, Fgt, Mgt, Pgt, dep_ref, dep_alt}, i = 1, 2, 3, …, n, (Pos, Fgt, Mgt, Pgt, dep_ref, dep_alt indicate the position, paternal genotype, maternal genotype, first child’s genotype, and number of reads supporting the reference or non-reference allele, respectively).

The hidden state is whether the haplotype transmitted to the fetus was the same as the first child, and was denoted as Q = {Hap0, Hap1}. The initial state distribution was defined as π = {1/2, 1/2}, as the lack of prior probability.

The emission probabilities matrix was represented using B = {bi,j},bi,j = P{Hap i|Nj}, i = 0, 1, and j = 1, 2, 3, …, n. The probabilities of Hap i transmitted to the fetus were calculated for given sites j using the following formula:

$$P\{Hapi|Nj\}=\frac{P\{Nj|Hapi\}\ast 0.5}{P\{Nj|Hap0\}\ast P\{Nj|Hap1\}\ast 0.5}$$

P{Nj| Hap0} and P{Nj |Hap1} were calculated using binomial distribution:

$${\rm{P}}\{{\rm{Nj}}|{\rm{Hap}}0\}={\rm{b}}({\rm{k}},\,{\rm{n}},\,{\rm{prob}}0)$$

$${\rm{P}}\{\mathrm{Nj}|\mathrm{Hap}1\}={\rm{b}}({\rm{k}},\,{\rm{n}},\,{\rm{prob}}1)$$

P {Nj| Hap i} is the probability of the observed state Nj at a given SNP loci j in the plasma when Hap i was transmitted to the fetus, k denotes the reads of the haplotype-rich allele, n refers to the total reads at a given site, and prob0 and prob1 represent the expected proportion of Hap 0 informative allele and Hap 1 informative alleles calculated according to the fetal DNA fraction, respectively, as illustrated in Supplemental Table S5.

The transition probabilities matrix was defined as *A* = {*a*{*i*}}, where

$$a\{i\}=(\begin{array}{cc}1-P & P\\ P & 1-P\end{array})\quad P=Pcomb\{i,i+1\}$$

*Pcomb* is the probability of recombination between two neighboring SNPs calculated by the genetic distance obtained from the hapmap.

Finally, the Viterbi algorithm^22^ was used to determine the most probable path through the observed data and deduce the inheritance of the maternal haplotype and paternal haplotype: r ∈ (0, 1). The fetal genotype was determined using the linkage information of the pathogenic mutation to the haplotype.

$$Path[i]=\sum _{j}^{n}ln(\max \,{b}_{i,j}\times ({a}_{i+1,r+1,j}))$$

### Accuracy of haplotype-based NIPT for HPA

Genomic DNA of the fetus was analyzed by Sanger sequencing in the hospital to validate the genotype determined by NIPT (Table 2) and short tandem repeat analysis on the maternal blood and amniotic fluid was conducted to exclude maternal contamination. The accuracy of haplotype-based NIPT of HPA was also evaluated by standard haplotypes obtained using sequencing data of amniotic fluids.

### Assessment of feasibility of NIPT in first-trimester

To investigate the effect of fetal DNA fraction and sequencing depth on the accuracy of the method, first, we evaluated the relationship between the different sequencing depth and the accuracy of the inferred fetal haplotype using computer simulation, meanwhile the fetal fraction was assigned to be 10%. Previous studies (Zhou *et al*.^16^; Xu *et al*.^17^) confirmed that the fetal DNA fraction at 10th gestational week was about 9% and the total increment of median fetal fraction from 5th to 12th gestational week was about just 1%; second, we also evaluate the relationship between fetal fraction/informative SNPs and the accuracy of the inferred the fetal haplotype and the sequencing depth was assigned to be 200X at the same time.

## Electronic supplementary material

Supplemental Materials
