# Supplementary material for: Haplotype-based Noninvasive Prenatal Diagnosis of Hyperphenylalaninemia through Targeted Sequencing of Maternal Plasma
Source: Sci Rep. 2018 Jan 9;8:161. doi: 10.1038/s41598-017-18358-y (PMC5760544; doi:10.1038/s41598-017-18358-y)
Supplement: Supplementary file 1 — Supplemental Materials [file 41598_2017_18358_MOESM1_ESM.pdf]

Supplementary information

Haplotype-based Noninvasive Prenatal Diagnosis of Hyperphenylalaninemia through Targeted Sequencing of Maternal Plasma

Jun Ye[1]*, Chao Chen [2, 3, 4]*, Yuan Yuan [2, 3, 4], Lianshu Han[1], Yaoshen Wang[2, 3, 4], Wenjuan Qiu[1], Huiwen Zhang[1], Asan[2, 3, 4+], Xuefan Gu[1+]

[1] Department of Pediatric Endocrinology and Genetic Metabolism, Shanghai Institute for Pediatric Research, Xinhua Hospital, Shanghai Jiao Tong University School of Medicine, Shanghai 200092, China;[2] Tianjin Translational Genomics Center, BGI-Tianjin, BGI-Shenzhen， Tianjin 300308, China;[3] Binhai Genomics Institute, BGI-Tianjin, BGI-Shenzhen, Tianjin 300308, China; [4] BGI-Shenzhen, Shenzhen, China; *These authors contributed equally to this work. [+] Correspondence and requests for materials should be addressed to X.G (email: guxuefan@xinhuamed.com.cn) or A. (email: 736971343@qq.com)

Supplementary figure and table legends

Supplementary Figure S1. Probability of Fetal Inherited Haplotype. X -axis represents the locus on chromosome 12 or 11, Y -axis represents the logarithm of the ratios of fetal different haplotype combinations. Red lines represent the fetus inherited paternal haplotype, the blue line fetus inherited from maternal haplotypes. The lines above zero (Cyan lines) indicate that the fetus inherited the pathogenic allele (Hap0), and the lines below zero indicate that the fetus inherited the benign allele (Hap1).

Supplementary Figure S2. Relationship between sequencing depth/ informative SNPs and the accuracy of the inferred fetal SNPs. X -axis represents the number of informative SNPs and Y -axis represents the sequencing depth. Color gradient chart represents error rate in logarithmic form.

Supplementary Figure S3. Relationship between fetal fraction/informative SNPs and the accuracy of the inferred maternal fetal SNPs. X-axis represents the number of informative SNPs and Y -axis represents the fetal fraction. Color gradient chart represents error rate in logarithmic form.

Supplementary Table S1. Statistics of Target Region Sequencing Data. Abbreviantions: $SNPs^{a}$ represents total SNPs in the customized probe; $SNPs^{b}$ represents SNPs in the family member in the targeted region; Phased SNPs infers to SNPs that were used to predict parental haplotypes with a trio strategy.

Supplementary Table S2. Accuracy of Haplotype-based NIPT for HPA. The accuracy of haplotype-based NIPT of HPA were evaluated by comparing to the standard haplotype obtained using sequencing data of parental and amniotic fluids samples. Abbreviantions: Loci N represents the consistent or inconsistent SNP number, RBR-N and CCE-N represents the inconsistent SNP number near the recombination breakpoint and Centromere or Chromosome edge respectively.

Supplementary Table S3. Relationship between sequencing depth/informative SNPs and the accuracy of the inferred maternal fetal SNPs.

A2, A3, A4, A5… A100 represents the number of informative SNPs and B1, C1, D1… AE are the sequencing depth. The other number is the accuracy of inferred maternal fetal SNPs.

Supplementary Table S4. Relationship between fetal fraction/informative SNPs and the accuracy of the inferred maternal fetal SNPs.

A2, A3, A4, A5… A100 represents the number of informative SNPs and B1, C1, D1… AE are fetal fraction. The other number is the inferred maternal fetal SNPs.

Supplementary Table S5. The emission probability for each phased SNP under different situation. The emission probabilities P {h0|Ni} and P {h1|Ni} given by binomial distribution were calculated for each phased. The emission probabilities matrix is B={bi,j}， bi,j=P{hi|Nj}, ,j=1, 2, 3,..., n.

Figure S1

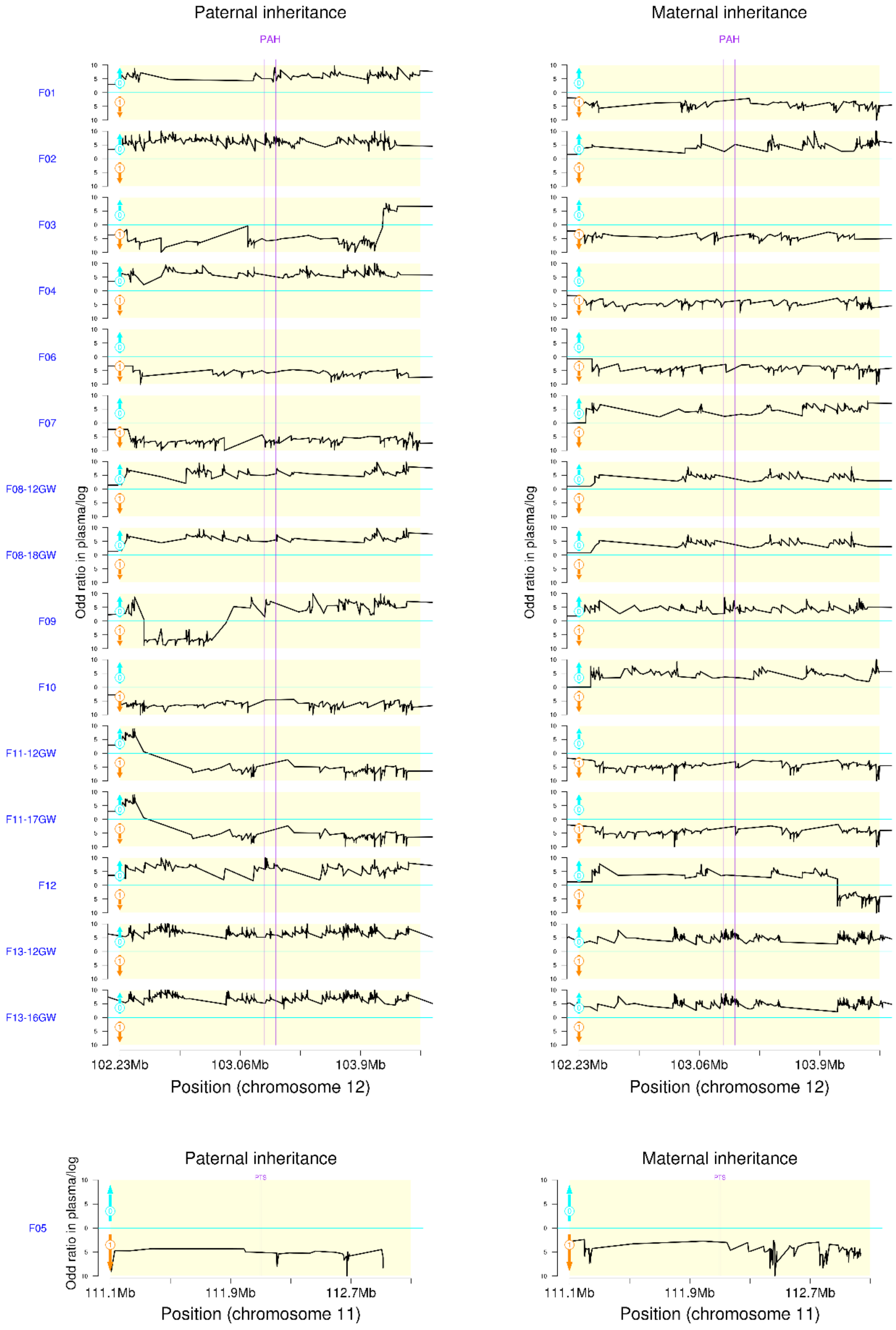

Figure S1. Probability of Fetal Inherited Haplotype.

Figure S2

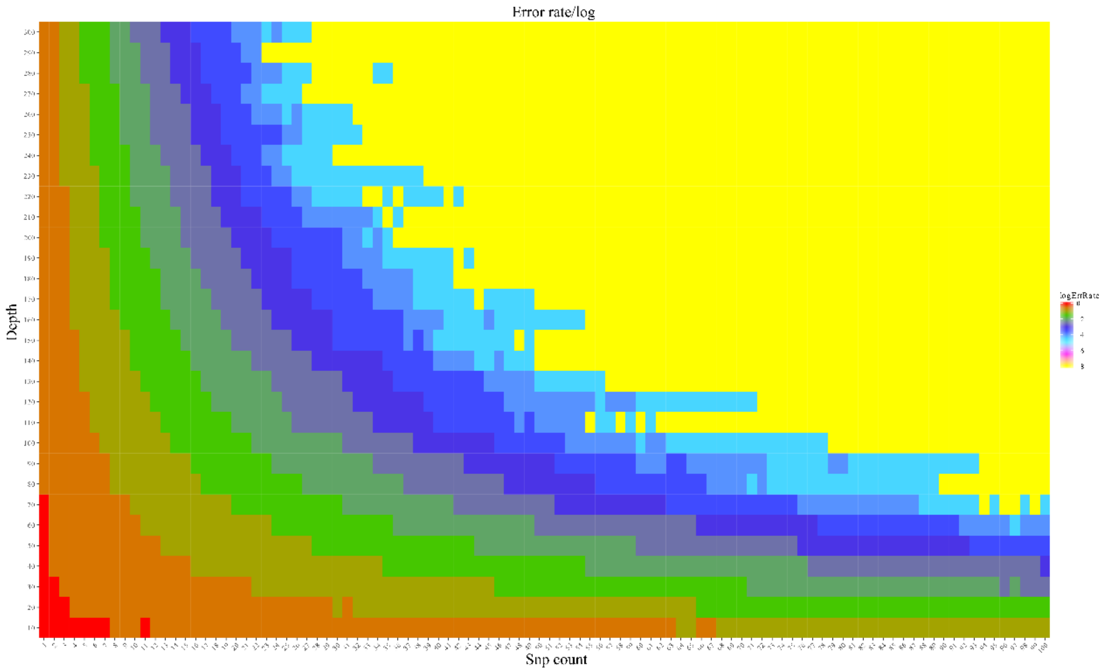

Figure S2. Relationship between sequencing depth/ informative SNPs and the accuracy of the inferred fetal SNPs. X -axis represents the number of informative SNPs and Y -axis represents the sequencing depth. Color gradient chart represents error rate in logarithmic form.

Figure S3

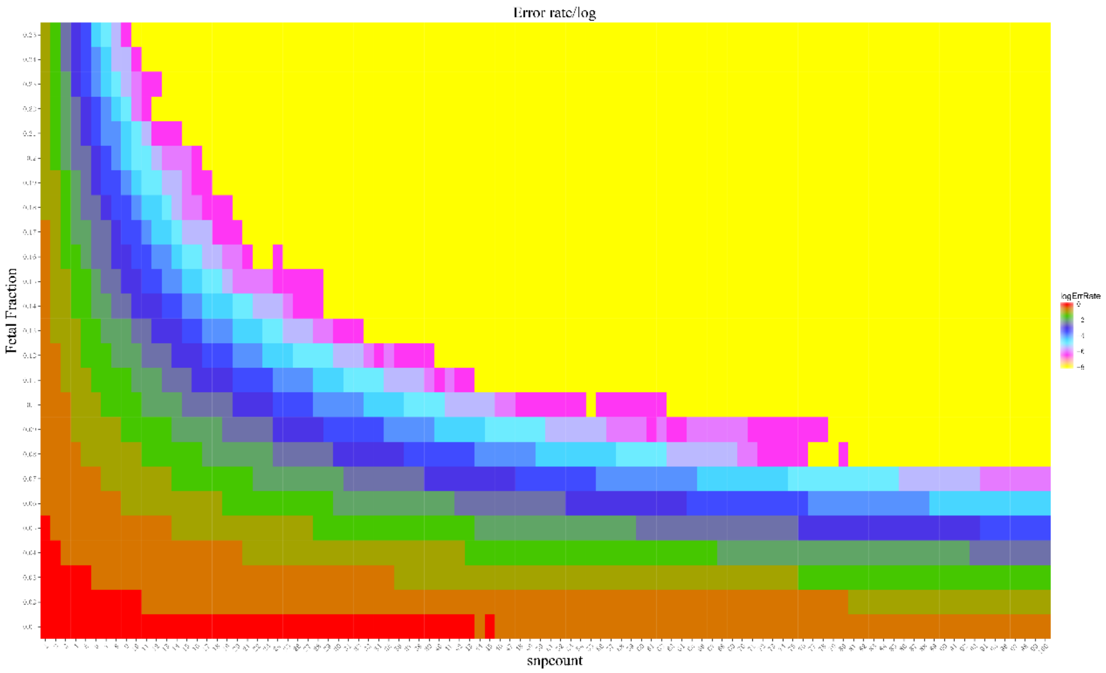

Supplementary Figure S3. Relationship between fetal fraction/informative SNPs and the accuracy of the inferred maternal fetal SNPs. X-axis represents the number of informative SNPs and Y -axis represents the fetal fraction. Color gradient chart represents error rate in logarithmic form.

Table S1

Pedigree	Sample ID	Data (Gb)	SNPs[a]	Targeted Region（PAH/PTS gene ±1M）				
				Reads	Mean Depth	Depth ≥20X (%)	SNPs[b]	Phased SNPs
F01	mother	1.97	18216	0.40	133.93	99.58%	905	223
	father	1.57	16532	0.38	127.78	99.48%	848	
	plasma	5.13	20796	1.16	282.63	99.93%	1,011	
	proband	1.61	17152	0.37	126.55	99.54%	849	
	amniotic fluid	1.77	16812	0.36	136.08	99.55%	916	
F02	mother	1.27	16293	0.32	110.19	99.43%	751	334
	father	2.48	17556	0.51	169.04	99.73%	845	
	plasma	6.30	22493	1.39	202.40	99.95%	1,013	
	proband	2.60	18384	0.56	183.11	99.75%	877	
	amniotic fluid	4.77	34954	0.95	206.61	99.66%	1,361	
F03	mother	2.01	14042	0.57	191.73	99.83%	759	167
	father	2.56	17758	0.49	161.74	99.77%	881	
	plasma	4.54	16011	1.34	297.33	99.96%	844	
	proband	2.49	17260	0.52	173.10	99.71%	757	
	amniotic fluid	4.64	32008	0.53	112.33	98.88%	1,162	
F04	mother	1.87	18002	0.33	125.56	99.57%	851	260
	father	2.74	19370	0.59	191.75	99.82%	864	
	plasma	6.43	24217	1.22	219.28	99.95%	1,078	
	proband	2.49	18031	0.53	175.80	99.76%	799	
	amniotic fluid	3.98	36254	0.43	77.27	97.74%	1,450	
F06	mother	1.96	19199	0.30	113.86	99.43%	828	236
	father	2.49	20250	0.38	142.13	99.56%	869	
	plasma	6.91	18308	2.06	443.09	99.94%	823	
	proband	1.90	19145	0.29	111.06	99.28%	777	
	amniotic fluid	6.09	34508	0.69	163.22	99.26%	1,340	
F07	mother	3.60	19454	0.58	269.52	99.82%	921	363
	father	2.97	16523	0.66	309.21	99.90%	911	
	plasma	12.08	28350	2.38	409.76	99.95%	1,239	
	proband	2.78	17215	0.61	281.75	99.86%	842	
	amniotic fluid	4.98	33959	1.21	245.62	99.67%	1,418	
F08	mother	1.38	15608	0.26	118.68	99.36%	746	236
	father	3.39	19134	0.59	277.07	99.81%	762	
	plasma-12GW	8.17	40264	2.46	399.45	99.90%	1,523	
	plasma-18GW	12.74	27284	2.34	426.87	99.94%	1,155	
	proband	3.44	20300	0.50	237.08	99.83%	900	
	amniotic fluid	6.59	34498	1.52	282.88	99.77%	1,367	

F09	mother	4.32	19252	0.69	318.95	99.88%	926	373
	father	3.81	19208	0.62	293.02	99.81%	927	
	plasma	8.80	21564	2.26	392.11	99.91%	1,038	
	proband	3.08	18957	0.53	247.33	99.77%	865	
	amniotic fluid	6.12	35295	1.34	272.19	99.74%	1,295	
F10	mother	2.97	17601	0.50	233.26	99.79%	866	318
	father	4.23	19155	0.72	331.41	99.83%	1,002	
	plasma	11.28	27386	2.14	395.03	99.95%	1,200	
	proband	4.24	17812	0.74	338.69	99.92%	939	
	amniotic fluid	4.64	33369	1.38	241.76	99.76%	1,532	
F11	mother	3.28	16641	0.69	318.83	99.81%	852	364
	father	4.30	18209	0.66	303.35	99.85%	957	
	plasma-12GW	9.70	43307	1.77	325.67	99.85%	1,614	
	plasma-17GW	11.92	27032	2.24	409.88	99.88%	1,213	
	proband	4.40	19691	0.72	326.68	99.86%	948	
	amniotic fluid	5.80	34178	1.40	232.40	99.71%	1,318	
F12	mother	4.22	21246	0.59	276.61	99.88%	892	326
	father	4.55	20576	0.69	314.88	99.85%	1,038	
	plasma	13.64	30015	2.49	590.63	99.78%	1,155	
	proband	4.89	21252	0.79	356.07	99.90%	935	
	amniotic fluid	6.65	37221	1.47	278.02	99.69%	1,507	
F13	mother	4.04	35689	0.82	160.11	99.11%	1,151	757
	father	4.16	34514	0.99	193.21	99.55%	1,301	
	plasma-12GW	12.06	35988	2.19	385.76	99.90%	1,154	
	plasma-16GW	5.26	35878	1.59	331.97	99.91%	1,151	
	proband	4.20	34891	0.60	181.17	99.15%	1,256	
F05	mother	0.94	16652	0.25	96.27	98.87%	749	121
	father	2.26	18658	0.52	174.72	99.75%	922	
	plasma	11.08	26629	2.26	223.77	99.93%	1,087	
	proband	2.75	19216	0.55	180.30	99.75%	923	
	amniotic fluid	5.85	34824	1.26	252.89	99.73%	1,364	

Table S1. Statistics of Target Region Sequencing Data. Abbreviantions: SNPs[a] represents total SNPs in the customized probe; SNPs[b] represents SNPs in the family member in the targeted region; Phased SNPs infers to SNPs that were used to predict parental haplotypes with a trio strategy.

Table S2

Family	Father				Mother				RBR-N	CCE-N
	Consistency		Non-consistency		Consistency		Non-consistency			
	Loci N	Percent	Loci N	Percent	Loci N	Percent	Loci N	Percent		
F01	117	1	0	0	98	1	0	0		0
F02	225	1	0	0	102	1	0	0	0	0
F03	106	1	0	0	84	1	0	0	0	0
F04	118	1	0	0	134	1	0	0	0	0
F05	75	1	0	0	66	1	0	0	0	0
F06	79	1	0	0	136	1	0	0	0	0
F07	250	1	0	0	104	1	0	0	0	0
F08-12GW	87	1	0	0	112	1	0	0	0	0
F08-18GW	120	1	0	0	108	1	0	0	0	0
F09	192	1	0	0	183	1	0	0	0	0
F10	194	1	0	0	118	1	0	0	0	0
F11-12GW	76	1	0	0	207	1	0	0	0	0
F11-17GW	158	1	0	0	188	1	0	0	0	0
F12	175	1	0	0	142	98.61%	2	1.39%	2	0

Table S2. Accuracy of Haplotype-based NIPT for HPA. The accuracy of haplotype-based NIPT of HPA were evaluated by comparing to the standard haplotype obtained using sequencing data of parental and amniotic fluids samples. Abbreviantions: Loci N represents the consistent or inconsistent SNP number, RBR-N and CCE-N represents the inconsistent SNP number near the recombination breakpoint and Centromere or Chromosome edge respectively.

Table S3

informative SNPs count\depth	10	20	30	40	50	60	70	80	90	100	110	120	130	140	150	160	170	180	190	200	210	220	230	240	250	260	270	280	290	300
1	0.62	0.59	0.57	0.68	0.66	0.65	0.64	0.71	0.70	0.69	0.68	0.74	0.73	0.72	0.72	0.76	0.75	0.75	0.74	0.78	0.78	0.77	0.77	0.80	0.79	0.79	0.79	0.82	0.81	0.81
2	0.59	0.68	0.65	0.71	0.69	0.74	0.72	0.76	0.75	0.78	0.77	0.80	0.79	0.82	0.81	0.83	0.82	0.84	0.84	0.85	0.85	0.86	0.86	0.87	0.87	0.88	0.88	0.89	0.88	0.90
3	0.57	0.65	0.70	0.74	0.72	0.75	0.78	0.80	0.79	0.80	0.83	0.85	0.83	0.85	0.86	0.87	0.87	0.88	0.89	0.90	0.89	0.90	0.91	0.91	0.91	0.92	0.92	0.93	0.93	0.93
4	0.68	0.71	0.74	0.76	0.78	0.80	0.82	0.83	0.84	0.85	0.86	0.88	0.88	0.89	0.90	0.90	0.91	0.92	0.92	0.92	0.93	0.93	0.94	0.94	0.95	0.95	0.95	0.96	0.96	0.96
5	0.66	0.69	0.72	0.78	0.79	0.81	0.82	0.85	0.86	0.86	0.87	0.90	0.90	0.91	0.91	0.93	0.93	0.93	0.94	0.95	0.95	0.95	0.95	0.96	0.96	0.96	0.97	0.97	0.97	0.97
6	0.65	0.74	0.75	0.80	0.81	0.85	0.85	0.87	0.88	0.89	0.90	0.92	0.92	0.93	0.93	0.94	0.94	0.95	0.95	0.96	0.96	0.97	0.97	0.97	0.97	0.98	0.98	0.98	0.98	0.98
7	0.64	0.72	0.78	0.81	0.82	0.85	0.87	0.89	0.89	0.90	0.92	0.93	0.93	0.94	0.95	0.96	0.96	0.96	0.97	0.97	0.97	0.97	0.98	0.98	0.98	0.98	0.98	0.99	0.99	0.99
8	0.71	0.76	0.80	0.83	0.85	0.88	0.89	0.90	0.92	0.92	0.93	0.95	0.95	0.96	0.96	0.97	0.97	0.97	0.98	0.98	0.98	0.98	0.98	0.99	0.99	0.99	0.99	0.99	0.99	0.99
9	0.70	0.75	0.79	0.84	0.86	0.88	0.89	0.92	0.93	0.93	0.94	0.96	0.96	0.96	0.97	0.97	0.98	0.98	0.98	0.98	0.98	0.99	0.99	0.99	0.99	0.99	0.99	0.99	0.99	0.99
10	0.69	0.78	0.81	0.85	0.87	0.90	0.91	0.93	0.93	0.94	0.95	0.96	0.96	0.97	0.97	0.98	0.98	0.98	0.99	0.99	0.99	0.99	0.99	0.99	0.99	0.99	0.99	1.00	1.00	1.00
11	0.68	0.77	0.82	0.86	0.88	0.91	0.92	0.94	0.94	0.95	0.96	0.97	0.97	0.98	0.98	0.98	0.98	0.99	0.99	0.99	0.99	0.99	0.99	0.99	1.00	1.00	1.00	1.00	1.00	1.00
12	0.74	0.80	0.84	0.87	0.90	0.92	0.93	0.94	0.95	0.96	0.97	0.97	0.98	0.98	0.98	0.99	0.99	0.99	0.99	0.99	0.99	0.99	1.00	1.00	1.00	1.00	1.00	1.00	1.00	1.00
13	0.73	0.79	0.83	0.88	0.90	0.92	0.93	0.95	0.96	0.96	0.97	0.98	0.98	0.98	0.99	0.99	0.99	0.99	0.99	0.99	1.00	1.00	1.00	1.00	1.00	1.00	1.00	1.00	1.00	1.00
14	0.72	0.82	0.85	0.89	0.91	0.94	0.94	0.96	0.96	0.97	0.98	0.98	0.98	0.99	0.99	0.99	0.99	0.99	0.99	1.00	1.00	1.00	1.00	1.00	1.00	1.00	1.00	1.00	1.00	1.00
15	0.72	0.81	0.86	0.90	0.91	0.94	0.95	0.96	0.97	0.97	0.98	0.98	0.99	0.99	0.99	0.99	0.99	1.00	1.00	1.00	1.00	1.00	1.00	1.00	1.00	1.00	1.00	1.00	1.00	1.00
16	0.76	0.83	0.87	0.90	0.93	0.95	0.96	0.96	0.97	0.98	0.98	0.99	0.99	0.99	0.99	0.99	1.00	1.00	1.00	1.00	1.00	1.00	1.00	1.00	1.00	1.00	1.00	1.00	1.00	1.00
17	0.75	0.82	0.87	0.91	0.93	0.95	0.96	0.97	0.98	0.98	0.98	0.99	0.99	0.99	0.99	1.00	1.00	1.00	1.00	1.00	1.00	1.00	1.00	1.00	1.00	1.00	1.00	1.00	1.00	1.00
18	0.75	0.84	0.88	0.92	0.93	0.96	0.96	0.97	0.98	0.98	0.99	0.99	0.99	1.00	0.99	1.00	1.00	1.00	1.00	1.00	1.00	1.00	1.00	1.00	1.00	1.00	1.00	1.00	1.00	1.00
19	0.74	0.84	0.89	0.92	0.94	0.96	0.97	0.98	0.98	0.98	0.99	0.99	0.99	1.00	1.00	1.00	1.00	1.00	1.00	1.00	1.00	1.00	1.00	1.00	1.00	1.00	1.00	1.00	1.00	1.00
20	0.78	0.85	0.90	0.93	0.95	0.96	0.97	0.98	0.98	0.99	0.99	0.99	0.99	1.00	1.00	1.00	1.00	1.00	1.00	1.00	1.00	1.00	1.00	1.00	1.00	1.00	1.00	1.00	1.00	1.00
21	0.77	0.85	0.89	0.93	0.95	0.96	0.97	0.98	0.99	0.99	0.99	0.99	1.00	1.00	1.00	1.00	1.00	1.00	1.00	1.00	1.00	1.00	1.00	1.00	1.00	1.00	1.00	1.00	1.00	1.00
22	0.77	0.86	0.90	0.93	0.95	0.97	0.98	0.98	0.99	0.99	0.99	1.00	1.00	1.00	1.00	1.00	1.00	1.00	1.00	1.00	1.00	1.00	1.00	1.00	1.00	1.00	1.00	1.00	1.00	1.00
23	0.77	0.86	0.91	0.94	0.95	0.97	0.98	0.98	0.99	0.99	0.99	1.00	1.00	1.00	1.00	1.00	1.00	1.00	1.00	1.00	1.00	1.00	1.00	1.00	1.00	1.00	1.00	1.00	1.00	1.00
24	0.80	0.87	0.92	0.94	0.96	0.98	0.98	0.99	0.99	0.99	1.00	1.00	1.00	1.00	1.00	1.00	1.00	1.00	1.00	1.00	1.00	1.00	1.00	1.00	1.00	1.00	1.00	1.00	1.00	1.00
25	0.80	0.87	0.91	0.95	0.96	0.98	0.98	0.99	0.99	0.99	1.00	1.00	1.00	1.00	1.00	1.00	1.00	1.00	1.00	1.00	1.00	1.00	1.00	1.00	1.00	1.00	1.00	1.00	1.00	1.00
26	0.79	0.88	0.92	0.95	0.96	0.98	0.98	0.99	0.99	0.99	1.00	1.00	1.00	1.00	1.00	1.00	1.00	1.00	1.00	1.00	1.00	1.00	1.00	1.00	1.00	1.00	1.00	1.00	1.00	1.00
27	0.79	0.88	0.92	0.95	0.97	0.98	0.99	0.99	0.99	0.99	1.00	1.00	1.00	1.00	1.00	1.00	1.00	1.00	1.00	1.00	1.00	1.00	1.00	1.00	1.00	1.00	1.00	1.00	1.00	1.00
28	0.81	0.89	0.93	0.95	0.97	0.98	0.99	0.99	0.99	1.00	1.00	1.00	1.00	1.00	1.00	1.00	1.00	1.00	1.00	1.00	1.00	1.00	1.00	1.00	1.00	1.00	1.00	1.00	1.00	1.00
29	0.81	0.89	0.93	0.96	0.97	0.98	0.99	0.99	1.00	1.00	1.00	1.00	1.00	1.00	1.00	1.00	1.00	1.00	1.00	1.00	1.00	1.00	1.00	1.00	1.00	1.00	1.00	1.00	1.00	1.00
30	0.81	0.90	0.93	0.96	0.97	0.99	0.99	0.99	1.00	1.00	1.00	1.00	1.00	1.00	1.00	1.00	1.00	1.00	1.00	1.00	1.00	1.00	1.00	1.00	1.00	1.00	1.00	1.00	1.00	1.00
31	0.80	0.89	0.94	0.96	0.97	0.99	0.99	0.99	1.00	1.00	1.00	1.00	1.00	1.00	1.00	1.00	1.00	1.00	1.00	1.00	1.00	1.00	1.00	1.00	1.00	1.00	1.00	1.00	1.00	1.00
32	0.83	0.91	0.94	0.96	0.98	0.99	0.99	0.99	1.00	1.00	1.00	1.00	1.00	1.00	1.00	1.00	1.00	1.00	1.00	1.00	1.00	1.00	1.00	1.00	1.00	1.00	1.00	1.00	1.00	1.00
33	0.83	0.90	0.94	0.97	0.98	0.99	0.99	1.00	1.00	1.00	1.00	1.00	1.00	1.00	1.00	1.00	1.00	1.00	1.00	1.00	1.00	1.00	1.00	1.00	1.00	1.00	1.00	1.00	1.00	1.00
34	0.82	0.91	0.94	0.97	0.98	0.99	0.99	1.00	1.00	1.00	1.00	1.00	1.00	1.00	1.00	1.00	1.00	1.00	1.00	1.00	1.00	1.00	1.00	1.00	1.00	1.00	1.00	1.00	1.00	1.00
35	0.82	0.91	0.95	0.97	0.98	0.99	0.99	1.00	1.00	1.00	1.00	1.00	1.00	1.00	1.00	1.00	1.00	1.00	1.00	1.00	1.00	1.00	1.00	1.00	1.00	1.00	1.00	1.00	1.00	1.00
36	0.84	0.92	0.95	0.97	0.98	0.99	0.99	1.00	1.00	1.00	1.00	1.00	1.00	1.00	1.00	1.00	1.00	1.00	1.00	1.00	1.00	1.00	1.00	1.00	1.00	1.00	1.00	1.00	1.00	1.00
37	0.84	0.91	0.95	0.97	0.98	0.99	0.99	1.00	1.00	1.00	1.00	1.00	1.00	1.00	1.00	1.00	1.00	1.00	1.00	1.00	1.00	1.00	1.00	1.00	1.00	1.00	1.00	1.00	1.00	1.00
38	0.84	0.92	0.95	0.98	0.98	0.99	1.00	1.00	1.00	1.00	1.00	1.00	1.00	1.00	1.00	1.00	1.00	1.00	1.00	1.00	1.00	1.00	1.00	1.00	1.00	1.00	1.00	1.00	1.00	1.00
39	0.83	0.92	0.96	0.98	0.99	0.99	1.00	1.00	1.00	1.00	1.00	1.00	1.00	1.00	1.00	1.00	1.00	1.00	1.00	1.00	1.00	1.00	1.00	1.00	1.00	1.00	1.00	1.00	1.00	1.00
40	0.85	0.93	0.96	0.98	0.99	0.99	1.00	1.00	1.00	1.00	1.00	1.00	1.00	1.00	1.00	1.00	1.00	1.00	1.00	1.00	1.00	1.00	1.00	1.00	1.00	1.00	1.00	1.00	1.00	1.00
41	0.85	0.92	0.96	0.98	0.99	0.99	1.00	1.00	1.00	1.00	1.00	1.00	1.00	1.00	1.00	1.00	1.00	1.00	1.00	1.00	1.00	1.00	1.00	1.00	1.00	1.00	1.00	1.00	1.00	1.00
42	0.85	0.93	0.96	0.98	0.99	1.00	1.00	1.00	1.00	1.00	1.00	1.00	1.00	1.00	1.00	1.00	1.00	1.00	1.00	1.00	1.00	1.00	1.00	1.00	1.00	1.00	1.00	1.00	1.00	1.00
43	0.84	0.93	0.96	0.98	0.99	1.00	1.00	1.00	1.00	1.00	1.00	1.00	1.00	1.00	1.00	1.00	1.00	1.00	1.00	1.00	1.00	1.00	1.00	1.00	1.00	1.00	1.00	1.00	1.00	1.00
44	0.86	0.94	0.97	0.98	0.99	1.00	1.00	1.00	1.00	1.00	1.00	1.00	1.00	1.00	1.00	1.00	1.00	1.00	1.00	1.00	1.00	1.00	1.00	1.00	1.00	1.00	1.00	1.00	1.00	1.00
45	0.86	0.93	0.97	0.98	0.99	1.00	1.00	1.00	1.00	1.00	1.00	1.00	1.00	1.00	1.00	1.00	1.00	1.00	1.00	1.00	1.00	1.00	1.00	1.00	1.00	1.00	1.00	1.00	1.00	1.00
46	0.86	0.94	0.97	0.98	0.99	1.00	1.00	1.00	1.00	1.00	1.00	1.00	1.00	1.00	1.00	1.00	1.00	1.00	1.00	1.00	1.00	1.00	1.00	1.00	1.00	1.00	1.00	1.00	1.00	1.00
47	0.86	0.94	0.97	0.99	0.99	1.00	1.00	1.00	1.00	1.00	1.00	1.00	1.00	1.00	1.00	1.00	1.00	1.00	1.00	1.00	1.00	1.00	1.00	1.00	1.00	1.00	1.00	1.00	1.00	1.00
48	0.87	0.94	0.97	0.99	0.99	1.00	1.00	1.00	1.00	1.00	1.00	1.00	1.00	1.00	1.00	1.00	1.00	1.00	1.00	1.00	1.00	1.00	1.00	1.00	1.00	1.00	1.00	1.00	1.00	1.00
49	0.87	0.94	0.97	0.99	0.99	1.00	1.00	1.00	1.00	1.00	1.00	1.00	1.00	1.00	1.00	1.00	1.00	1.00	1.00	1.00	1.00	1.00	1.00	1.00	1.00	1.00	1.00	1.00	1.00	1.00
50	0.87	0.95	0.97	0.99	0.99	1.00	1.00	1.00	1.00	1.00	1.00	1.00	1.00	1.00	1.00	1.00	1.00	1.00	1.00	1.00	1.00	1.00	1.00	1.00	1.00	1.00	1.00	1.00	1.00	1.00
51	0.87	0.95	0.98	0.99	0.99	1.00	1.00	1.00	1.00	1.00	1.00	1.00	1.00	1.00	1.00	1.00	1.00	1.00	1.00	1.00	1.00	1.00	1.00	1.00	1.00	1.00	1.00	1.00	1.00	1.00
52	0.88	0.95	0.98	0.99	1.00	1.00	1.00	1.00	1.00	1.00	1.00	1.00	1.00	1.00	1.00	1.00	1.00	1.00	1.00	1.00	1.00	1.00	1.00	1.00	1.00	1.00	1.00	1.00	1.00	1.00
53	0.88	0.95	0.98	0.99	0.99	1.00	1.00	1.00	1.00	1.00	1.00	1.00	1.00	1.00	1.00	1.00	1.00	1.00	1.00	1.00	1.00	1.00	1.00	1.00	1.00	1.00	1.00	1.00	1.00	1.00
54	0.88	0.95	0.98	0.99	1.00	1.00	1.00	1.00	1.00	1.00	1.00	1.00	1.00	1.00	1.00	1.00	1.00	1.00	1.00	1.00	1.00	1.00	1.00	1.00	1.00	1.00	1.00	1.00	1.00	1.00
55	0.88	0.95	0.98	0.99	1.00	1.00	1.00	1.00	1.00	1.00	1.00	1.00	1.00	1.00	1.00	1.00	1.00	1.00	1.00	1.00	1.00	1.00	1.00	1.00	1.00	1.00	1.00	1.00	1.00	1.00
56	0.89	0.96	0.98	0.99	1.00	1.00	1.00	1.00	1.00	1.00	1.00	1.00	1.00	1.00	1.00	1.00	1.00	1.00	1.00	1.00	1.00	1.00	1.00	1.00	1.00	1.00	1.00	1.00	1.00	1.00
57	0.89	0.95	0.98	0.99	1.00	1.00	1.00	1.00	1.00	1.00	1.00	1.00	1.00	1.00	1.00	1.00	1.00	1.00	1.00	1.00	1.00	1.00	1.00	1.00	1.00	1.00	1.00	1.00	1.00	1.00
58	0.89	0.96	0.98	0.99	1.00	1.00	1.00	1.00	1.00	1.00	1.00	1.00	1.00	1.00	1.00	1.00	1.00	1.00	1.00	1.00	1.00	1.00	1.00	1.00	1.00	1.00	1.00	1.00	1.00	1.00
59	0.88	0.96	0.98	0.99	1.00	1.00	1.00	1.00	1.00	1.00	1.00	1.00	1.00	1.00	1.00	1.00	1.00	1.00	1.00	1.00	1.00	1.00	1.00	1.00	1.00	1.00	1.00	1.00	1.00	1.00
60	0.90	0.96	0.98	0.99	1.00	1.00	1.00	1.00	1.00	1.00	1.00	1.00	1.00	1.00	1.00	1.00	1.00	1.00	1.00	1.00	1.00	1.00	1.00	1.00	1.00	1.00	1.00	1.00	1.00	1.00
61	0.90	0.96	0.98	0.99	1.00	1.00	1.00	1.00	1.00	1.00	1.00	1.00	1.00	1.00	1.00	1.00	1.00	1.00	1.00	1.00	1.00	1.00	1.00	1.00	1.00	1.00	1.00	1.00	1.00	1.00
62	0.89	0.96	0.98	0.99	1.00	1.00	1.00	1.00	1.00	1.00	1.00	1.00	1.00	1.00	1.00	1.00	1.00	1.00	1.00	1.00	1.00	1.00	1.00	1.00	1.00	1.00	1.00	1.00	1.00	1.00
63	0.89	0.96	0.99	0.99	1.00	1.00	1.00	1.00	1.00	1.00	1.00	1.00	1.00	1.00	1.00	1.00	1.00	1.00	1.00	1.00	1.00	1.00	1.00	1.00	1.00	1.00	1.00	1.00	1.00	1.00
64	0.90	0.97	0.99	0.99	1.00	1.00	1.00	1.00	1.00	1.00	1.00	1.00	1.00	1.00	1.00	1.00	1.00	1.00	1.00	1.00	1.00	1.00	1.00	1.00	1.00	1.00	1.00	1.00	1.00	1.00
65	0.90	0.96	0.99	0.99	1.00	1.00	1.00	1.00	1.00	1.00	1.00	1.00	1.00	1.00	1.00	1.00	1.00	1.00	1.00	1.00	1.00	1.00	1.00	1.00	1.00	1.00	1.00	1.00	1.00	1.00
66	0.90	0.97	0.99	1.00	1.00	1.00	1.00	1.00	1.00	1.00	1.00	1.00	1.00	1.00	1.00	1.00	1.00	1.00	1.00	1.00	1.00	1.00	1.00	1.00	1.00	1.00	1.00	1.00	1.00	1.00
67	0.90	0.97	0.99	1.00	1.00	1.00	1.00	1.00	1.00	1.00	1.00	1.00	1.00	1.00	1.00	1.00	1.00	1.00	1.00	1.00	1.00	1.00	1.00	1.00	1.00	1.00	1.00	1.00	1.00	1.00
68	0.91	0.97	0.99	1.00	1.00	1.00	1.00	1.00	1.00	1.00	1.00	1.00	1.00	1.00	1.00	1.00	1.00	1.00	1.00	1.00	1.00	1.00	1.00	1.00	1.00	1.00	1.00	1.00	1.00	1.00
69	0.91	0.97	0.99	1.00	1.00	1.00	1.00	1.00	1.00	1.00	1.00	1.00	1.00	1.00	1.00	1.00	1.00	1.00	1.00	1.00	1.00	1.00	1.00	1.00	1.00	1.00	1.00	1.00	1.00	1.00
70	0.91	0.97	0.99	1.00	1.00	1.00	1.00	1.00	1.00	1.00	1.00	1.00	1.00	1.00	1.00	1.00	1.00	1.00	1.00	1.00	1.00	1.00	1.00	1.00	1.00	1.00	1.00	1.00	1.00	1.00
71	0.91	0.97	0.99	1.00	1.00	1.00	1.00	1.00	1.00	1.00	1.00	1.00	1.00	1.00	1.00	1.00	1.00	1.00	1.00	1.00	1.00	1.00	1.00	1.00	1.00	1.00	1.00	1.00	1.00	1.00
72	0.92	0.97	0.99	1.00	1.00	1.00	1.00	1.00	1.00	1.00	1.00	1.00	1.00	1.00	1.00	1.00	1.00	1.00	1.00	1.00	1.00	1.00	1.00	1.00	1.00	1.00	1.00	1.00	1.00	1.00
73	0.92	0.97	0.99	1.00	1.00	1.00	1.00	1.00	1.00	1.00	1.00	1.00	1.00	1.00	1.00	1.00	1.00	1.00	1.00	1.00	1.00	1.00	1.00	1.00	1.00	1.00	1.00	1.00	1.00	1.00
74	0.91	0.97	0.99	1.00	1.00	1.00	1.00	1.00	1.00	1.00	1.00	1.00	1.00	1.00	1.00	1.00	1.00	1.00	1.00	1.00	1.00	1.00	1.00	1.00	1.00	1.00	1.00	1.00	1.00	1.00
75	0.91	0.97	0.99	1.00	1.00	1.00	1.00	1.00	1.00	1.00	1.00	1.00	1.00	1.00	1.00	1.00	1.00	1.00	1.00	1.00	1.00	1.00	1.00	1.00	1.00	1.00	1.00	1.00	1.00	1.00
76	0.92	0.98	0.99	1.00	1.00	1.00	1.00	1.00	1.00	1.00	1.00	1.00	1.00	1.00	1.00	1.00	1.00	1.00	1.00	1.00	1.00	1.00	1.00	1.00	1.00	1.00	1.00	1.00	1.00	1.00
77	0.92	0.98	0.99	1.00	1.00	1.00	1.00	1.00	1.00	1.00	1.00	1.00	1.00	1.00	1.00	1.00	1.00	1.00	1.00	1.00	1.00	1.00	1.00	1.00	1.00	1.00	1.00	1.00	1.00	1.00
78	0.92	0.98	0.99	1.00	1.00	1.00	1.00	1.00	1.00	1.00	1.00	1.00	1.00	1.00	1.00	1.00	1.00	1.00	1.00	1.00	1.00	1.00	1.00	1.00	1.00	1.00	1.00	1.00	1.00	1.00
79	0.92	0.98	0.99	1.00	1.00	1.00	1.00	1.00	1.00	1.00	1.00	1.00	1.00	1.00	1.00	1.00	1.00	1.00	1.00	1.00	1.00	1.00	1.00	1.00	1.00	1.00	1.00	1.00	1.00	1.00
80	0.93	0.98	0.99	1.00	1.00	1.00	1.00	1.00	1.00	1.00	1.00	1.00	1.00	1.00	1.00	1.00	1.00	1.00	1.00	1.00	1.00	1.00	1.00	1.00	1.00	1.00	1.00	1.00	1.00	1.00
81	0.93	0.98	0.99	1.00	1.00	1.00	1.00	1.00	1.00	1.00	1.00	1.00	1.00	1.00	1.00	1.00	1.00	1.00	1.00	1.00	1.00	1.00	1.00	1.00	1.00	1.00	1.00	1.00	1.00	1.00
82	0.93	0.98	0.99	1.00	1.00	1.00	1.00	1.00	1.00	1.00	1.00	1.00	1.00	1.00	1.00	1.00	1.00	1.00	1.00	1.00	1.00	1.00	1.00	1.00	1.00	1.00	1.00	1.00	1.00	1.00
83	0.92	0.98	0.99	1.00	1.00	1.00	1.00	1.00	1.00	1.00	1.00	1.00	1.00	1.00	1.00	1.00	1.00	1.00	1.00	1.00	1.00	1.00	1.00	1.00	1.00	1.00	1.00	1.00	1.00	1.00
84	0.93	0.98	0.99	1.00	1.00	1.00	1.00	1.00	1.00	1.00	1.00	1.00	1.00	1.00	1.00	1.00	1.00	1.00	1.00	1.00	1.00	1.00	1.00	1.00	1.00	1.00	1.00	1.00	1.00	1.00
85	0.93	0.98	0.99	1.00	1.00	1.00	1.00	1.00	1.00	1.00	1.00	1.00	1.00	1.00	1.00	1.00	1.00	1.00	1.00	1.00	1.00	1.00	1.00	1.00	1.00	1.00	1.00	1.00	1.00	1.00
86	0.93	0.98	0.99	1.00	1.00	1.00	1.00	1.00	1.00	1.00	1.00	1.00	1.00	1.00	1.00	1.00	1.00	1.00	1.00	1.00	1.00	1.00	1.00	1.00	1.00	1.00	1.00	1.00	1.00	1.00
87	0.93	0.98	0.99	1.00	1.00	1.00	1.00	1.00	1.00	1.00	1.00	1.00	1.00	1.00	1.00	1.00	1.00	1.00	1.00	1.00	1.00	1.00	1.00	1.00	1.00	1.00	1.00	1.00	1.00	1.00
88	0.94	0.98	1.00	1.00	1.00	1.00	1.00	1.00	1.00	1.00	1.00	1.00	1.00	1.00	1.00	1.00	1.00	1.00	1.00	1.00	1.00	1.00	1.00	1.00	1.00	1.00	1.00	1.00	1.00	1.00
89	0.94	0.98	0.99	1.00	1.00	1.00	1.00	1.00	1.00	1.00	1.00	1.00	1.00	1.00	1.00	1.00	1.00	1.00	1.00	1.00	1.00	1.00	1.00	1.00	1.00	1.00	1.00	1.00	1.00	1.00
90	0.93	0.98	1.00	1.00	1.00	1.00	1.00	1.00	1.00	1.00	1.00	1.00	1.00	1.00	1.00	1.00	1.00	1.00	1.00	1.00	1.00	1.00	1.00	1.00	1.00	1.00	1.00	1.00	1.00	1.00
91	0.93	0.98	1.00	1.00	1.00	1.00	1.00	1.00	1.00	1.00	1.00	1.00	1.00	1.00	1.00	1.00	1.00	1.00	1.00	1.00	1.00	1.00	1.00	1.00	1.00	1.00	1.00	1.00	1.00	1.00
92	0.94	0.99	1.00	1.00	1.00	1.00	1.00	1.00	1.00	1.00	1.00	1.00	1.00	1.00	1.00	1.00	1.00	1.00	1.00	1.00	1.00	1.00	1.00	1.00	1.00	1.00	1.00	1.00	1.00	1.00
93	0.94	0.98	1.00	1.00	1.00	1.00	1.00	1.00	1.00	1.00	1.00	1.00	1.00	1.00	1.00	1.00	1.00	1.00	1.00	1.00	1.00	1.00	1.00	1.00	1.00	1.00	1.00	1.00	1.00	1.00
94	0.94	0.99	1.00	1.00	1.00	1.00	1.00	1.00	1.00	1.00	1.00	1.00	1.00	1.00	1.00	1.00	1.00	1.00	1.00	1.00	1.00	1.00	1.00	1.00	1.00	1.00	1.00	1.00	1.00	1.00
95	0.94	0.99	1.00	1.00	1.00	1.00	1.00	1.00	1.00	1.00	1.00	1.00	1.00	1.00	1.00	1.00	1.00	1.00	1.00	1.00	1.00	1.00	1.00	1.00	1.00	1.00	1.00	1.00	1.00	1.00
96	0.94	0.99	1.00	1.00	1.00	1.00	1.00	1.00	1.00	1.00	1.00	1.00	1.00	1.00	1.00	1.00	1.00	1.00	1.00	1.00	1.00	1.00	1.00	1.00	1.00	1.00	1.00	1.00	1.00	1.00
97	0.94	0.99	1.00	1.00	1.00	1.00	1.00	1.00	1.00	1.00	1.00	1.00	1.00	1.00	1.00	1.00	1.00	1.00	1.00	1.00	1.00	1.00	1.00	1.00	1.00	1.00	1.00	1.00	1.00	1.00
98	0.94	0.99	1.00	1.00	1.00	1.00	1.00	1.00	1.00	1.00	1.00	1.00	1.00	1.00	1.00	1.00	1.00	1.00	1.00	1.00	1.00	1.00	1.00	1.00	1.00	1.00	1.00	1.00	1.00	1.00
99	0.94	0.99	1.00	1.00	1.00	1.00	1.00	1.00	1.00	1.00	1.00	1.00	1.00	1.00	1.00	1.00	1.00	1.00	1.00	1.00	1.00	1.00	1.00	1.00	1.00	1.00	1.00	1.00	1.00	1.00
100	0.95	0.99	1.00	1.00	1.00	1.00	1.00	1.00	1.00	1.00	1.00	1.00	1.00	1.00	1.00	1.00	1.00	1.00	1.00	1.00	1.00	1.00	1.00	1.00	1.00	1.00	1.00	1.00	1.00	1.00

Table S4

informative SNPs count\	1%	2%	3%	4%	5%	6%	7%	8%	9%	10%	11%	12%	13%	14%	15%	16%	17%	18%	19%	20%	21%	22%	23%	24%	25%
**1**	0.53	0.58	0.58	0.64	0.64	0.69	0.69	0.74	0.74	0.78	0.78	0.82	0.82	0.86	0.86	0.89	0.89	0.91	0.91	0.93	0.93	0.95	0.95	0.96	0.96
**2**	0.56	0.60	0.64	0.67	0.71	0.74	0.77	0.80	0.83	0.85	0.87	0.89	0.91	0.92	0.94	0.95	0.96	0.96	0.97	0.97	0.98	0.98	0.98	0.99	0.99
**3**	0.55	0.61	0.64	0.70	0.73	0.78	0.80	0.85	0.86	0.90	0.91	0.93	0.94	0.96	0.96	0.98	0.98	0.99	0.99	0.99	0.99	1.00	1.00	1.00	1.00
**4**	0.57	0.62	0.68	0.73	0.77	0.81	0.85	0.88	0.90	0.93	0.94	0.96	0.97	0.98	0.98	0.99	0.99	0.99	1.00	1.00	1.00	1.00	1.00	1.00	1.00
**5**	0.56	0.64	0.68	0.75	0.79	0.84	0.87	0.90	0.92	0.95	0.96	0.97	0.98	0.99	0.99	0.99	1.00	1.00	1.00	1.00	1.00	1.00	1.00	1.00	1.00
**6**	0.58	0.65	0.71	0.76	0.81	0.86	0.89	0.92	0.94	0.96	0.97	0.98	0.99	0.99	0.99	1.00	1.00	1.00	1.00	1.00	1.00	1.00	1.00	1.00	1.00
**7**	0.57	0.66	0.71	0.78	0.83	0.87	0.90	0.94	0.95	0.97	0.98	0.99	0.99	1.00	1.00	1.00	1.00	1.00	1.00	1.00	1.00	1.00	1.00	1.00	1.00
**8**	0.59	0.66	0.73	0.80	0.85	0.89	0.92	0.95	0.97	0.98	0.99	0.99	1.00	1.00	1.00	1.00	1.00	1.00	1.00	1.00	1.00	1.00	1.00	1.00	1.00
**9**	0.58	0.67	0.74	0.81	0.86	0.90	0.93	0.96	0.97	0.98	0.99	0.99	1.00	1.00	1.00	1.00	1.00	1.00	1.00	1.00	1.00	1.00	1.00	1.00	1.00
**10**	0.60	0.68	0.76	0.82	0.87	0.91	0.94	0.96	0.98	0.99	0.99	1.00	1.00	1.00	1.00	1.00	1.00	1.00	1.00	1.00	1.00	1.00	1.00	1.00	1.00
**11**	0.59	0.69	0.76	0.83	0.88	0.92	0.95	0.97	0.98	0.99	0.99	1.00	1.00	1.00	1.00	1.00	1.00	1.00	1.00	1.00	1.00	1.00	1.00	1.00	1.00
**12**	0.60	0.70	0.78	0.84	0.89	0.93	0.96	0.98	0.99	0.99	1.00	1.00	1.00	1.00	1.00	1.00	1.00	1.00	1.00	1.00	1.00	1.00	1.00	1.00	1.00
**13**	0.60	0.70	0.78	0.85	0.90	0.94	0.96	0.98	0.99	0.99	1.00	1.00	1.00	1.00	1.00	1.00	1.00	1.00	1.00	1.00	1.00	1.00	1.00	1.00	1.00
**14**	0.61	0.71	0.79	0.86	0.91	0.95	0.97	0.98	0.99	1.00	1.00	1.00	1.00	1.00	1.00	1.00	1.00	1.00	1.00	1.00	1.00	1.00	1.00	1.00	1.00
**15**	0.61	0.71	0.79	0.87	0.91	0.95	0.97	0.99	0.99	1.00	1.00	1.00	1.00	1.00	1.00	1.00	1.00	1.00	1.00	1.00	1.00	1.00	1.00	1.00	1.00
**16**	0.62	0.72	0.81	0.87	0.92	0.96	0.98	0.99	0.99	1.00	1.00	1.00	1.00	1.00	1.00	1.00	1.00	1.00	1.00	1.00	1.00	1.00	1.00	1.00	1.00
**17**	0.61	0.73	0.81	0.88	0.93	0.96	0.98	0.99	1.00	1.00	1.00	1.00	1.00	1.00	1.00	1.00	1.00	1.00	1.00	1.00	1.00	1.00	1.00	1.00	1.00
**18**	0.62	0.73	0.82	0.89	0.94	0.97	0.98	0.99	1.00	1.00	1.00	1.00	1.00	1.00	1.00	1.00	1.00	1.00	1.00	1.00	1.00	1.00	1.00	1.00	1.00
**19**	0.62	0.74	0.82	0.89	0.94	0.97	0.98	0.99	1.00	1.00	1.00	1.00	1.00	1.00	1.00	1.00	1.00	1.00	1.00	1.00	1.00	1.00	1.00	1.00	1.00
**20**	0.63	0.74	0.83	0.90	0.94	0.97	0.99	0.99	1.00	1.00	1.00	1.00	1.00	1.00	1.00	1.00	1.00	1.00	1.00	1.00	1.00	1.00	1.00	1.00	1.00
**21**	0.63	0.75	0.83	0.91	0.95	0.98	0.99	1.00	1.00	1.00	1.00	1.00	1.00	1.00	1.00	1.00	1.00	1.00	1.00	1.00	1.00	1.00	1.00	1.00	1.00
**22**	0.64	0.75	0.84	0.91	0.95	0.98	0.99	1.00	1.00	1.00	1.00	1.00	1.00	1.00	1.00	1.00	1.00	1.00	1.00	1.00	1.00	1.00	1.00	1.00	1.00
**23**	0.63	0.76	0.85	0.91	0.96	0.98	0.99	1.00	1.00	1.00	1.00	1.00	1.00	1.00	1.00	1.00	1.00	1.00	1.00	1.00	1.00	1.00	1.00	1.00	1.00
**24**	0.64	0.76	0.85	0.92	0.96	0.98	0.99	1.00	1.00	1.00	1.00	1.00	1.00	1.00	1.00	1.00	1.00	1.00	1.00	1.00	1.00	1.00	1.00	1.00	1.00
**25**	0.64	0.77	0.86	0.92	0.96	0.98	0.99	1.00	1.00	1.00	1.00	1.00	1.00	1.00	1.00	1.00	1.00	1.00	1.00	1.00	1.00	1.00	1.00	1.00	1.00
**26**	0.65	0.77	0.86	0.93	0.97	0.99	0.99	1.00	1.00	1.00	1.00	1.00	1.00	1.00	1.00	1.00	1.00	1.00	1.00	1.00	1.00	1.00	1.00	1.00	1.00
**27**	0.64	0.77	0.86	0.93	0.97	0.99	0.99	1.00	1.00	1.00	1.00	1.00	1.00	1.00	1.00	1.00	1.00	1.00	1.00	1.00	1.00	1.00	1.00	1.00	1.00
**28**	0.65	0.78	0.87	0.93	0.97	0.99	1.00	1.00	1.00	1.00	1.00	1.00	1.00	1.00	1.00	1.00	1.00	1.00	1.00	1.00	1.00	1.00	1.00	1.00	1.00
**29**	0.65	0.78	0.87	0.94	0.97	0.99	1.00	1.00	1.00	1.00	1.00	1.00	1.00	1.00	1.00	1.00	1.00	1.00	1.00	1.00	1.00	1.00	1.00	1.00	1.00
**30**	0.66	0.78	0.88	0.94	0.97	0.99	1.00	1.00	1.00	1.00	1.00	1.00	1.00	1.00	1.00	1.00	1.00	1.00	1.00	1.00	1.00	1.00	1.00	1.00	1.00
**31**	0.65	0.79	0.88	0.94	0.98	0.99	1.00	1.00	1.00	1.00	1.00	1.00	1.00	1.00	1.00	1.00	1.00	1.00	1.00	1.00	1.00	1.00	1.00	1.00	1.00
**32**	0.66	0.79	0.89	0.95	0.98	0.99	1.00	1.00	1.00	1.00	1.00	1.00	1.00	1.00	1.00	1.00	1.00	1.00	1.00	1.00	1.00	1.00	1.00	1.00	1.00
**33**	0.66	0.80	0.89	0.95	0.98	0.99	1.00	1.00	1.00	1.00	1.00	1.00	1.00	1.00	1.00	1.00	1.00	1.00	1.00	1.00	1.00	1.00	1.00	1.00	1.00
**34**	0.66	0.80	0.89	0.95	0.98	0.99	1.00	1.00	1.00	1.00	1.00	1.00	1.00	1.00	1.00	1.00	1.00	1.00	1.00	1.00	1.00	1.00	1.00	1.00	1.00
**35**	0.66	0.80	0.90	0.95	0.98	0.99	1.00	1.00	1.00	1.00	1.00	1.00	1.00	1.00	1.00	1.00	1.00	1.00	1.00	1.00	1.00	1.00	1.00	1.00	1.00
**36**	0.67	0.81	0.90	0.96	0.98	0.99	1.00	1.00	1.00	1.00	1.00	1.00	1.00	1.00	1.00	1.00	1.00	1.00	1.00	1.00	1.00	1.00	1.00	1.00	1.00
**37**	0.67	0.81	0.90	0.96	0.98	1.00	1.00	1.00	1.00	1.00	1.00	1.00	1.00	1.00	1.00	1.00	1.00	1.00	1.00	1.00	1.00	1.00	1.00	1.00	1.00
**38**	0.67	0.81	0.91	0.96	0.99	1.00	1.00	1.00	1.00	1.00	1.00	1.00	1.00	1.00	1.00	1.00	1.00	1.00	1.00	1.00	1.00	1.00	1.00	1.00	1.00
**39**	0.67	0.81	0.91	0.96	0.99	1.00	1.00	1.00	1.00	1.00	1.00	1.00	1.00	1.00	1.00	1.00	1.00	1.00	1.00	1.00	1.00	1.00	1.00	1.00	1.00
**40**	0.68	0.82	0.91	0.96	0.99	1.00	1.00	1.00	1.00	1.00	1.00	1.00	1.00	1.00	1.00	1.00	1.00	1.00	1.00	1.00	1.00	1.00	1.00	1.00	1.00
**41**	0.68	0.82	0.91	0.97	0.99	1.00	1.00	1.00	1.00	1.00	1.00	1.00	1.00	1.00	1.00	1.00	1.00	1.00	1.00	1.00	1.00	1.00	1.00	1.00	1.00
**42**	0.68	0.82	0.92	0.97	0.99	1.00	1.00	1.00	1.00	1.00	1.00	1.00	1.00	1.00	1.00	1.00	1.00	1.00	1.00	1.00	1.00	1.00	1.00	1.00	1.00
**43**	0.68	0.83	0.92	0.97	0.99	1.00	1.00	1.00	1.00	1.00	1.00	1.00	1.00	1.00	1.00	1.00	1.00	1.00	1.00	1.00	1.00	1.00	1.00	1.00	1.00
**44**	0.68	0.83	0.92	0.97	0.99	1.00	1.00	1.00	1.00	1.00	1.00	1.00	1.00	1.00	1.00	1.00	1.00	1.00	1.00	1.00	1.00	1.00	1.00	1.00	1.00
**45**	0.68	0.83	0.92	0.97	0.99	1.00	1.00	1.00	1.00	1.00	1.00	1.00	1.00	1.00	1.00	1.00	1.00	1.00	1.00	1.00	1.00	1.00	1.00	1.00	1.00
**46**	0.69	0.83	0.93	0.97	0.99	1.00	1.00	1.00	1.00	1.00	1.00	1.00	1.00	1.00	1.00	1.00	1.00	1.00	1.00	1.00	1.00	1.00	1.00	1.00	1.00
**47**	0.69	0.84	0.93	0.97	0.99	1.00	1.00	1.00	1.00	1.00	1.00	1.00	1.00	1.00	1.00	1.00	1.00	1.00	1.00	1.00	1.00	1.00	1.00	1.00	1.00
**48**	0.69	0.84	0.93	0.98	0.99	1.00	1.00	1.00	1.00	1.00	1.00	1.00	1.00	1.00	1.00	1.00	1.00	1.00	1.00	1.00	1.00	1.00	1.00	1.00	1.00
**49**	0.69	0.84	0.93	0.98	0.99	1.00	1.00	1.00	1.00	1.00	1.00	1.00	1.00	1.00	1.00	1.00	1.00	1.00	1.00	1.00	1.00	1.00	1.00	1.00	1.00
**50**	0.70	0.84	0.93	0.98	0.99	1.00	1.00	1.00	1.00	1.00	1.00	1.00	1.00	1.00	1.00	1.00	1.00	1.00	1.00	1.00	1.00	1.00	1.00	1.00	1.00
**51**	0.69	0.85	0.94	0.98	0.99	1.00	1.00	1.00	1.00	1.00	1.00	1.00	1.00	1.00	1.00	1.00	1.00	1.00	1.00	1.00	1.00	1.00	1.00	1.00	1.00
**52**	0.70	0.85	0.94	0.98	0.99	1.00	1.00	1.00	1.00	1.00	1.00	1.00	1.00	1.00	1.00	1.00	1.00	1.00	1.00	1.00	1.00	1.00	1.00	1.00	1.00
**53**	0.70	0.85	0.94	0.98	1.00	1.00	1.00	1.00	1.00	1.00	1.00	1.00	1.00	1.00	1.00	1.00	1.00	1.00	1.00	1.00	1.00	1.00	1.00	1.00	1.00
**54**	0.70	0.85	0.94	0.98	1.00	1.00	1.00	1.00	1.00	1.00	1.00	1.00	1.00	1.00	1.00	1.00	1.00	1.00	1.00	1.00	1.00	1.00	1.00	1.00	1.00
**55**	0.70	0.86	0.94	0.98	1.00	1.00	1.00	1.00	1.00	1.00	1.00	1.00	1.00	1.00	1.00	1.00	1.00	1.00	1.00	1.00	1.00	1.00	1.00	1.00	1.00
**56**	0.71	0.86	0.94	0.98	1.00	1.00	1.00	1.00	1.00	1.00	1.00	1.00	1.00	1.00	1.00	1.00	1.00	1.00	1.00	1.00	1.00	1.00	1.00	1.00	1.00
**57**	0.70	0.86	0.95	0.98	1.00	1.00	1.00	1.00	1.00	1.00	1.00	1.00	1.00	1.00	1.00	1.00	1.00	1.00	1.00	1.00	1.00	1.00	1.00	1.00	1.00
**58**	0.71	0.86	0.95	0.98	1.00	1.00	1.00	1.00	1.00	1.00	1.00	1.00	1.00	1.00	1.00	1.00	1.00	1.00	1.00	1.00	1.00	1.00	1.00	1.00	1.00
**59**	0.71	0.86	0.95	0.99	1.00	1.00	1.00	1.00	1.00	1.00	1.00	1.00	1.00	1.00	1.00	1.00	1.00	1.00	1.00	1.00	1.00	1.00	1.00	1.00	1.00
**60**	0.71	0.87	0.95	0.99	1.00	1.00	1.00	1.00	1.00	1.00	1.00	1.00	1.00	1.00	1.00	1.00	1.00	1.00	1.00	1.00	1.00	1.00	1.00	1.00	1.00
**61**	0.71	0.87	0.95	0.99	1.00	1.00	1.00	1.00	1.00	1.00	1.00	1.00	1.00	1.00	1.00	1.00	1.00	1.00	1.00	1.00	1.00	1.00	1.00	1.00	1.00
**62**	0.71	0.87	0.95	0.99	1.00	1.00	1.00	1.00	1.00	1.00	1.00	1.00	1.00	1.00	1.00	1.00	1.00	1.00	1.00	1.00	1.00	1.00	1.00	1.00	1.00
**63**	0.71	0.87	0.95	0.99	1.00	1.00	1.00	1.00	1.00	1.00	1.00	1.00	1.00	1.00	1.00	1.00	1.00	1.00	1.00	1.00	1.00	1.00	1.00	1.00	1.00
**64**	0.72	0.87	0.96	0.99	1.00	1.00	1.00	1.00	1.00	1.00	1.00	1.00	1.00	1.00	1.00	1.00	1.00	1.00	1.00	1.00	1.00	1.00	1.00	1.00	1.00
**65**	0.72	0.88	0.96	0.99	1.00	1.00	1.00	1.00	1.00	1.00	1.00	1.00	1.00	1.00	1.00	1.00	1.00	1.00	1.00	1.00	1.00	1.00	1.00	1.00	1.00
**66**	0.72	0.88	0.96	0.99	1.00	1.00	1.00	1.00	1.00	1.00	1.00	1.00	1.00	1.00	1.00	1.00	1.00	1.00	1.00	1.00	1.00	1.00	1.00	1.00	1.00
**67**	0.72	0.88	0.96	0.99	1.00	1.00	1.00	1.00	1.00	1.00	1.00	1.00	1.00	1.00	1.00	1.00	1.00	1.00	1.00	1.00	1.00	1.00	1.00	1.00	1.00
**68**	0.72	0.88	0.96	0.99	1.00	1.00	1.00	1.00	1.00	1.00	1.00	1.00	1.00	1.00	1.00	1.00	1.00	1.00	1.00	1.00	1.00	1.00	1.00	1.00	1.00
**69**	0.72	0.88	0.96	0.99	1.00	1.00	1.00	1.00	1.00	1.00	1.00	1.00	1.00	1.00	1.00	1.00	1.00	1.00	1.00	1.00	1.00	1.00	1.00	1.00	1.00
**70**	0.73	0.88	0.96	0.99	1.00	1.00	1.00	1.00	1.00	1.00	1.00	1.00	1.00	1.00	1.00	1.00	1.00	1.00	1.00	1.00	1.00	1.00	1.00	1.00	1.00
**71**	0.72	0.89	0.96	0.99	1.00	1.00	1.00	1.00	1.00	1.00	1.00	1.00	1.00	1.00	1.00	1.00	1.00	1.00	1.00	1.00	1.00	1.00	1.00	1.00	1.00
**72**	0.73	0.89	0.96	0.99	1.00	1.00	1.00	1.00	1.00	1.00	1.00	1.00	1.00	1.00	1.00	1.00	1.00	1.00	1.00	1.00	1.00	1.00	1.00	1.00	1.00
**73**	0.73	0.89	0.97	0.99	1.00	1.00	1.00	1.00	1.00	1.00	1.00	1.00	1.00	1.00	1.00	1.00	1.00	1.00	1.00	1.00	1.00	1.00	1.00	1.00	1.00
**74**	0.73	0.89	0.97	0.99	1.00	1.00	1.00	1.00	1.00	1.00	1.00	1.00	1.00	1.00	1.00	1.00	1.00	1.00	1.00	1.00	1.00	1.00	1.00	1.00	1.00
**75**	0.73	0.89	0.97	0.99	1.00	1.00	1.00	1.00	1.00	1.00	1.00	1.00	1.00	1.00	1.00	1.00	1.00	1.00	1.00	1.00	1.00	1.00	1.00	1.00	1.00
**76**	0.73	0.89	0.97	0.99	1.00	1.00	1.00	1.00	1.00	1.00	1.00	1.00	1.00	1.00	1.00	1.00	1.00	1.00	1.00	1.00	1.00	1.00	1.00	1.00	1.00
**77**	0.73	0.89	0.97	0.99	1.00	1.00	1.00	1.00	1.00	1.00	1.00	1.00	1.00	1.00	1.00	1.00	1.00	1.00	1.00	1.00	1.00	1.00	1.00	1.00	1.00
**78**	0.74	0.90	0.97	0.99	1.00	1.00	1.00	1.00	1.00	1.00	1.00	1.00	1.00	1.00	1.00	1.00	1.00	1.00	1.00	1.00	1.00	1.00	1.00	1.00	1.00
**79**	0.74	0.90	0.97	0.99	1.00	1.00	1.00	1.00	1.00	1.00	1.00	1.00	1.00	1.00	1.00	1.00	1.00	1.00	1.00	1.00	1.00	1.00	1.00	1.00	1.00
**80**	0.74	0.90	0.97	0.99	1.00	1.00	1.00	1.00	1.00	1.00	1.00	1.00	1.00	1.00	1.00	1.00	1.00	1.00	1.00	1.00	1.00	1.00	1.00	1.00	1.00
**81**	0.74	0.90	0.97	0.99	1.00	1.00	1.00	1.00	1.00	1.00	1.00	1.00	1.00	1.00	1.00	1.00	1.00	1.00	1.00	1.00	1.00	1.00	1.00	1.00	1.00
**82**	0.74	0.90	0.97	0.99	1.00	1.00	1.00	1.00	1.00	1.00	1.00	1.00	1.00	1.00	1.00	1.00	1.00	1.00	1.00	1.00	1.00	1.00	1.00	1.00	1.00
**83**	0.74	0.90	0.97	1.00	1.00	1.00	1.00	1.00	1.00	1.00	1.00	1.00	1.00	1.00	1.00	1.00	1.00	1.00	1.00	1.00	1.00	1.00	1.00	1.00	1.00
**84**	0.74	0.90	0.97	1.00	1.00	1.00	1.00	1.00	1.00	1.00	1.00	1.00	1.00	1.00	1.00	1.00	1.00	1.00	1.00	1.00	1.00	1.00	1.00	1.00	1.00
**85**	0.74	0.91	0.97	1.00	1.00	1.00	1.00	1.00	1.00	1.00	1.00	1.00	1.00	1.00	1.00	1.00	1.00	1.00	1.00	1.00	1.00	1.00	1.00	1.00	1.00
**86**	0.75	0.91	0.98	1.00	1.00	1.00	1.00	1.00	1.00	1.00	1.00	1.00	1.00	1.00	1.00	1.00	1.00	1.00	1.00	1.00	1.00	1.00	1.00	1.00	1.00
**87**	0.75	0.91	0.98	1.00	1.00	1.00	1.00	1.00	1.00	1.00	1.00	1.00	1.00	1.00	1.00	1.00	1.00	1.00	1.00	1.00	1.00	1.00	1.00	1.00	1.00
**88**	0.75	0.91	0.98	1.00	1.00	1.00	1.00	1.00	1.00	1.00	1.00	1.00	1.00	1.00	1.00	1.00	1.00	1.00	1.00	1.00	1.00	1.00	1.00	1.00	1.00
**89**	0.75	0.91	0.98	1.00	1.00	1.00	1.00	1.00	1.00	1.00	1.00	1.00	1.00	1.00	1.00	1.00	1.00	1.00	1.00	1.00	1.00	1.00	1.00	1.00	1.00
**90**	0.75	0.91	0.98	1.00	1.00	1.00	1.00	1.00	1.00	1.00	1.00	1.00	1.00	1.00	1.00	1.00	1.00	1.00	1.00	1.00	1.00	1.00	1.00	1.00	1.00
**91**	0.75	0.91	0.98	1.00	1.00	1.00	1.00	1.00	1.00	1.00	1.00	1.00	1.00	1.00	1.00	1.00	1.00	1.00	1.00	1.00	1.00	1.00	1.00	1.00	1.00
**92**	0.75	0.91	0.98	1.00	1.00	1.00	1.00	1.00	1.00	1.00	1.00	1.00	1.00	1.00	1.00	1.00	1.00	1.00	1.00	1.00	1.00	1.00	1.00	1.00	1.00
**93**	0.75	0.92	0.98	1.00	1.00	1.00	1.00	1.00	1.00	1.00	1.00	1.00	1.00	1.00	1.00	1.00	1.00	1.00	1.00	1.00	1.00	1.00	1.00	1.00	1.00
**94**	0.76	0.92	0.98	1.00	1.00	1.00	1.00	1.00	1.00	1.00	1.00	1.00	1.00	1.00	1.00	1.00	1.00	1.00	1.00	1.00	1.00	1.00	1.00	1.00	1.00
**95**	0.76	0.92	0.98	1.00	1.00	1.00	1.00	1.00	1.00	1.00	1.00	1.00	1.00	1.00	1.00	1.00	1.00	1.00	1.00	1.00	1.00	1.00	1.00	1.00	1.00
**96**	0.76	0.92	0.98	1.00	1.00	1.00	1.00	1.00	1.00	1.00	1.00	1.00	1.00	1.00	1.00	1.00	1.00	1.00	1.00	1.00	1.00	1.00	1.00	1.00	1.00
**97**	0.76	0.92	0.98	1.00	1.00	1.00	1.00	1.00	1.00	1.00	1.00	1.00	1.00	1.00	1.00	1.00	1.00	1.00	1.00	1.00	1.00	1.00	1.00	1.00	1.00
**98**	0.76	0.92	0.98	1.00	1.00	1.00	1.00	1.00	1.00	1.00	1.00	1.00	1.00	1.00	1.00	1.00	1.00	1.00	1.00	1.00	1.00	1.00	1.00	1.00	1.00
**99**	0.76	0.92	0.98	1.00	1.00	1.00	1.00	1.00	1.00	1.00	1.00	1.00	1.00	1.00	1.00	1.00	1.00	1.00	1.00	1.00	1.00	1.00	1.00	1.00	1.00
**100**	0.76	0.92	0.98	1.00	1.00	1.00	1.00	1.00	1.00	1.00	1.00	1.00	1.00	1.00	1.00	1.00	1.00	1.00	1.00	1.00	1.00	1.00	1.00	1.00	1.00

Table S5

Paternal genotype	Maternal genotype	Fetal genotype	P{h0|Ni}	P{h1|Ni}
0/0	0/1	0/0	P{Aratio=(1-e)/2|Ni}	P{Aratio=1/2|Ni}
0/0	0/1	0/1	P{Aratio=1/2|Ni}	P{Aratio=(1-e)/2|Ni}
1/1	0/1	0/1	P{Aratio=1/2|Ni}	P{Aratio=(1+e)/2|Ni}
1/1	0/1	1/1	P{Aratio=(1+e)/2|Ni}	P{Aratio=1/2|Ni}
0/1	0/0	0/0	P{Aratio=0|Ni}	P{Aratio=e/2|Ni}
0/1	0/0	0/1	P{Aratio=e/2|Ni}	P{Aratio=0|Ni}
0/1	1/1	0/1	P{Aratio=1-e/2|Ni}	P{Aratio=1|Ni}
0/1	1/1	1/1	P{Aratio=1|Ni}	P{Aratio=1-e/2|Ni}

Table S5. The emission probability for each phased SNP under different situation.
The emission probabilities P {h0|Ni} and P {h1|Ni} given by binomial distribution were calculated for each phased. The emission probabilities matrix is B={bi,j}， bi,j=P{hi|Nj}, ,j=1, 2, 3,..., n.
